# Impact of the Post-Transplant Period and Lifestyle Diseases on Human Gut Microbiota in Kidney Graft Recipients

**DOI:** 10.3390/microorganisms8111724

**Published:** 2020-11-04

**Authors:** Nessrine Souai, Oumaima Zidi, Amor Mosbah, Imen Kosai, Jameleddine El Manaa, Naima Bel Mokhtar, Elias Asimakis, Panagiota Stathopoulou, Ameur Cherif, George Tsiamis, Soumaya Kouidhi

**Affiliations:** 1 Laboratory of Biotechnology and Valorisation of Bio-GeoRessources, Higher Institute of Biotechnology of Sidi Thabet, BiotechPole of Sidi Thabet, University of Manouba, Ariana 2020, Tunisia; nessrine.souai@fst.utm.tn (N.S.); oumaima.zidi@hotmail.fr (O.Z.); amor.mosbah@gmail.com (A.M.); ameur.cherif@uma.tn (A.C.); 2 Department of Biology, Faculty of Sciences of Tunis, University of Tunis El Manar, Farhat Hachad Universitary Campus, Rommana 1068, Tunis, Tunisia; 3 Unit of Organ Transplant Military Training Hospital, Mont Fleury 1008, Tunis, Tunisia; imenelkosai@gmail.com (I.K.); Manaajamel@yahoo.fr (J.E.M.); 4 Laboratory of Systems Microbiology and Applied Genomics, Department of Environmental Engineering, University of Patras, 2 Seferi St, 30100 Agrinio, Greece; naima1503@gmail.com (N.B.M.); eliasasim@gmail.com (E.A.); panayotastathopoulou@gmail.com (P.S.); gtsiamis@upatras.gr (G.T.)

**Keywords:** 16S rRNA gene, amplicon sequencing, dysbiosis, bioinformatics, kidney transplantation

## Abstract

Gaining long-term graft function and patient life quality remain critical challenges following kidney transplantation. Advances in immunology, gnotobiotics, and culture-independent molecular techniques have provided growing insights into the complex relationship of the microbiome and the host. However, little is known about the over time-shift of the gut microbiota in the context of kidney transplantation and its impact on both graft and health stability. Here we aimed to characterize the structure of gut microbiota within stable kidney graft recipients. We enrolled forty kidney transplant patients after at least three months of transplantation and compared them to eighteen healthy controls. The overall microbial community structure of the kidney transplanted group was clearly different from control subjects. We found lower relative abundances of Actinobacteria, Bacteroidetes, and Verrucomicrobia within the patient group and a higher abundance of Proteobacteria compared to the control group. Both richness and Shannon diversity indexes were significantly lower in the kidney graft recipients than in healthy controls. Post-graft period was positively correlated with the relative abundance of the Proteobacteria phylum, especially *Escherichia.Shigella* genus. Interestingly, only *Parabacteroides* was found to significantly differentiate patients that were not suffering from lifestyle diseases and those who suffer from post-graft complications. Furthermore, network analysis showed that the occurrence of lifestyle diseases was significantly linked with a higher number of negative interactions of *Sutterella* and *Succinivibrio* genera within patients. This study characterizes gut microbiome fluctuation in stable kidney transplant patients after a long post-allograft period. Analysis of fecal microbiota could be useful for nephrologists as a new clinical tool that can improve kidney allograft monitoring and outcomes.

## 1. Introduction

Increasing kidney disease and subsequent chronic kidney disease (CKD) is related to the ageing society and high morbidity due to lifestyle diseases such as diabetes, atherosclerosis, and hypertension [1]. During the past decade, kidney transplantation was increasingly recognized as the treatment of choice for medically suitable patients with CKD [2]. As well as improving quality of life, successful transplantation confers significant benefits by improving the morbidity and mortality of CKD patients who receive kidney transplant over those who undergo dialysis [3]. The clinical concern of the successful transplant patient is rejection. At five-years posttransplant, kidney allograft survival is as low as 71% [4]. Standards of care protocols recommend regular surveillance for detecting and treating early rejection, which is done by checking creatinine and urine proteinuria and/or by routine biopsy at regular posttransplant intervals [5]. However, a recent study proved that 41.3% of kidney recipients have been receiving low-value, unnecessary biopsies [6]. This evidence suggests that better diagnostic, non-invasive tools may be more effective than invasive, costly biopsies in the context of predicting kidney rejection. In addition, dosing of immunosuppressors to establish therapeutic levels in recipients of organ transplants remains a challenging task because of high interpatient and intrapatient variability in drug metabolism. Tacrolimus possesses a narrow therapeutic index with sub-therapeutic levels leading to immune rejection and supra-therapeutic levels that could lead to nephrotoxicity and neurotoxicity [7]. Altogether, these studies suggest that nephrologists and transplant patients need better tests than creatinine and proteinuria and less invasive approaches than routine biopsies to determine when transplant patients should be investigated for rejection and immunosuppressive treatment.

Both human and mouse studies reported that the gut microbial community is associated with complications in kidney allograft recipients, including overall survival, infections, and graft rejection [8,9]. Growing evidence suggests that the gut microbiota serves as both the origin and the target of post-transplant complications. Several factors pre-, intra-, and/or post-transplantation can result in an altered microbiome and consequent dysbiosis. These factors include the use of antimicrobials and immunosuppressant drugs, hemodialysis, and the new post-surgical anatomy. Dysbiosis may lead to several post-transplantation complications such as the risk of infection (urinary tract infection, infectious diarrhea), adverse immunologic phenomena (autoimmune hemolytic anemia), graft rejection, and increased mortality rates. Selectively avoiding these alterations and inducing eubiotic changes in the peri-transplantation setting may hold preventative and therapeutic potential [10].

Additionally, the microbiota has been found to modulate drug pharmacokinetics and accordingly, therapeutic response. Of interest, deep sequencing identified that the abundance of *Faecalibacterium prausnitzii* in feces early after kidney transplantation is associated with tacrolimus dosing requirements in kidney transplant recipients [11]. Furthermore, some studies investigated the potential of intestinal microbial flora as microbial biomarkers for the non-invasive diagnosis and selection of appropriate personalized treatment for CKD [12]. However, it remains unclear which bacterial genera are optimal to predict post graft complications in the kidney transplant population. Therefore, this study was performed in order to identify and understand changes in gut microbial composition between stable kidney transplant patients and healthy controls. 

## 2. Materials and Methods 

### 2.1. Study Cohort

With the approval of the Tunisian Military University hospital’s ethics committee, we enrolled forty renal transplant recipients for a fecal specimen collection and clinical data study. The subjects provided the fecal specimens within one day of production, and the samples were frozen at −80 °C. Similarly, stool samples were collected from (n = 18) healthy subjects and written informed consent was obtained for each enrollee.

In order to investigate the variability in fecal gut microbiota over time, we divided the study cohort into three subgroups according to the graft stability state: short post-graft period (“SG”: from 3 months to 1 year; n = 11), medium-length post-graft period (“MG” from 1 year to 10 years; n = 20), and long post-transplant period (“LG” from 10 to 22 years n = 9). These three subgroups were compared to the healthy control samples (n = 18). We conducted further analysis of the fecal specimens based on the health status of the participants and whether or not they suffer from any lifestyle diseases that were named associated diseases (ADs). In the present study, n = 24 patients suffered from one or multiple complications from the following list: obesity, diabetes, high blood pressure, and dyslipidemia. However, n = 16 were kidney graft recipients that did not suffer from other associated diseases. Age and gender factors were also investigated but not reported in the present study for non-significance. This could be due to the small number of subjects covering a broad range of ages. 

The study was conducted according to the principles expressed in the Declaration of Helsinki, and all research procedures were approved on 5 March 2018 by the Bioethics Committee of the Military University Hospital of Tunis (No. 05032018).

### 2.2. DNA Extraction, First-Step PCR Amplification and Purification

Total genomic DNA was isolated from the fecal specimens using an InnuPREP DNA kit (Analytik Jena, Jena, Germany) according to the manufacturer’s instructions. Three replicates of each sample were extracted. The quality and quantity of DNA samples were tested using a Q5000 micro-volume UV–Vis spectrophotometer (Quawell Technology, San Jose, CA, USA). DNA samples were stored in Eppendorf tubes at −20 °C until PCR amplification and amplicon sequencing analysis. PCR was performed with a KAPA Taq PCR kit (KAPA Biosystems, Wilmington, MA, USA) and the previously extracted DNA as a template. The variable V3–V4 region of the bacterial 16S rRNA gene was amplified with the primer pair U341F-MiSeq 5-CCTACG GGR SGC AGC AG-3 and 805R-MiSeq 5-GA CTACHV GGG TAT CTA ATC C-3 [13]. Each 25 µL reaction contained 2.5 µL of KAPA buffer (10×), 0.2 µL of dNTPs solution (25 mM), 1 µL of each primer solution (10 µM), 0.12 µL of KAPA Taq DNA polymerase solution (5 U/µL), and 1 µL of the template DNA solution, and was finalized with 19.18 µL of sterile deionized water. The PCR amplifications were performed with a 3 min incubation at 95 °C followed by 35 cycles of 95 °C for 30 s, 53 °C for 30 s, and 72 °C for 1 min, and a final 5 min extension at 72 °C. Negative and positive controls were always performed in parallel. All PCR products were separated in a 1.5% (wt/vol) agarose gel in TAE buffer (40 mM Tris-acetate, 1 mM EDTA). The desired, approximately 550 bp amplification product was visualized in Bio-Rad’s Gel Doc XR+ system. Positive PCR products were purified from unincorporated primers and nucleotides with a 20% PEG, 2.5 M NaCl solution, and centrifuged at 14,000× *g* for 20 min. The precipitate was washed twice with 125 µL of a 70% vol/vol ethanol solution and centrifuged at 14,000× *g* for 10 min. The dried precipitates were suspended in 15 µL of sterile deionized water, and the concentration was measured with a Q5000 micro-volume UV–Vis spectrophotometer (Quawell Technology, San Jose, CA, USA).

### 2.3. Second-Step PCR Amplification (Indexing) and Purification 

The resulting PCR amplicons were diluted up to 10 ng/µL and then used as templates within the second-step PCR for further amplification, and to include the indexes (barcodes) as well as the Illumina adaptors. The combinatorial use of index primers resulted in unique samples that were pooled and sequenced by Macrogen using a 2 × 300 bp pair-end kit on a MiSeq platform.

In more detail, the amplification reaction was performed using the KAPA HiFi HotStart PCR kit in a final volume of 50 µL. Each reaction contained 10 µL of KAPA HiFi Fidelity buffer (5×), 1.5 µL of dNTPs solution (10 mM each), 5 µL of the forward indexing primer (10 µM), 5 µL of the reverse indexing primer (10 µM), 1 µL of KAPA HiFi Hot Start DNA polymerase (1 U/µL), 2 µL from the diluted PCR product (10 ng/µL), and 25.5 µL of sterile deionized water. The PCR amplifications were performed with a 3-min incubation at 95 °C followed by eight cycles of 95 °C for 30 s, 55 °C for 30 s, and 72 °C for 30 s, and a final 5-min terminator reaction at 72 °C. The resulting amplicons from indexing PCR were purified using the NucleoMag NGS Clean-up and Size Selection kit (Macherey-Nagel, Deuren, Germany) according to the manufacturer’s recommendations. Amplicons from different samples were quantified with a Quawell Q5000 micro-volume UV–Vis spectrophotometer and merged in equimolar ratios (8 nM). 

### 2.4. Bioinformatic and Statistical Analysis

Raw sequencing reads were de-multiplexed and converted to FASTQ, and the Illumina adapters were trimmed using Illumina standard algorithms. Paired-end reads were assembled, trimmed by length, and further corrected for error and quality using the usearch -fastq_mergepairs option. All subsequent analyses were conducted in usearch version v.10 [14]. Briefly, the quality of the assembled sequences was further improved using the -fastq_filter, followed by finding unique read sequences and abundances by using the -fastx_uniques option. Sequences were clustered into operational taxonomic units (OTUs) using the -cluster_otus command [15]. Chimeras were removed using the -unoise3 option of usearch v.10 [16]. Taxonomy was assigned using the syntax option against the SILVA 128 release database [17,18].

Relative abundance plots were created based on the OTU table along with the taxonomy data previously obtained with the aim to visualize the most dominant OTUs in each sample group at phylum and genus levels. Alpha diversity was then computed based on the MicrobioSeq package to analyze within-group diversity [19]. Significant differences between groups were calculated using pair-wise ANOVA [20].

Principal coordinate analysis (PCA) was performed on the resulting distance matrix to search for similarities between control and KT groups. To estimate the variation of gut bacteria among period subgroups, short, medium, and long period (SG, MG, and LG), and associated disease (AD) and no AD subgroups compared to healthy controls, a canonical analysis of principal coordinates (CAP) using PERMANOVA+ [21], was performed. In the last analysis, Bray–Curtis similarity index with 999 permutations and 0.05 of significance was applied. Square root transformation was applied to each the data matrix. All tests were performed using PRIMER 6+ software and the PERMANOVA+ version of this program [21].

Furthermore, we aimed to investigate possible interactions between microorganisms. These interactions were analyzed and visualized through the co-occurrence analyses and association network. This may correspond to microorganisms performing similar or complementary functions and/or sharing similar preferred environmental conditions, but not necessarily having physical interactions [22,23]. Co-occurrence network analysis of the main OTUs was performed using the CoNet plugin [24] in Cytoscape 3.8.1 (Institute for system biology, Seattle, WA, USA), and co-occurrence profiles were obtained using Gephi 0.9.2 (Gephi, WebAtlas, Paris, France). To build the network, an ensemble of the Pearson and Spearman correlation coefficients, Mutual Information, and the Bray–Curtis and Kullback–Leibler dissimilarity indices were combined. To compute the statistical significance of the copresence/mutual exclusion, edge-specific permutation and bootstrap score distributions were calculated with 1000 iterations. Edges with original scores outside the 0.95 range of their bootstrap distribution were discarded, and *p*-values were multiple-testing corrected using the Benjamini–Hochberg method. Nodes in each network visualization correspond to microbial OTUs and edges to the microbial associations. The size of each node is proportional to the degree of interactions. The Bar chart graphs were performed using the ggplot2. 3.3.2 (RStudio, Houston, TX, USA) package in R version 4.0.3 (The R Foundation for Statistical Computing, Vienna, Austria) to indicate the number of positive and negative interaction for each bacterial member of the gut microbiota. 

Finally, serial group comparison analysis was performed to compare variables among the study groups of samples and to detect differences in composition and abundance using the non-parametric Kruskal–Wallis rank sum test along with the Wilcoxon rank sum test [25].

## 3. Results

### 3.1. Kidney Transplant Cohort 

Kidney transplant individuals were divided into 12 women and 28 men aged between 17 and 67 years. Demographic and alpha diversity data of the kidney transplant cohort are further listed in Supplementary Table S1. A total dataset of 1,027,064 reads was obtained. The results reported herein are based on the computation of genomic data obtained from the 40 patients’ fecal specimens and 18 healthy samples. We subdivided our patient cohort into subgroups according to the graft period (SG, MG, and LG) and the presence or absence of associated diseases (AD and no AD, respectively). Age and gender were also considered but they were not found as major factors to significantly highlight the shift in the gut bacteria following kidney transplantation. Overall, we found five main phyla that were abundant in the study groups, namely Bacteroidetes, Firmicutes, Proteobacteria, Verrucomicrobia, and Actinobacteria. *Prevotella 9*, in the Bacteroidetes phylum, as well as *Faecalibacterium* were the bacterial groups with the highest abundance of this cohort. 

### 3.2. Structure of the Gut Bacterial Community of the Kidney Transplanted Patients

**Figure 1:**
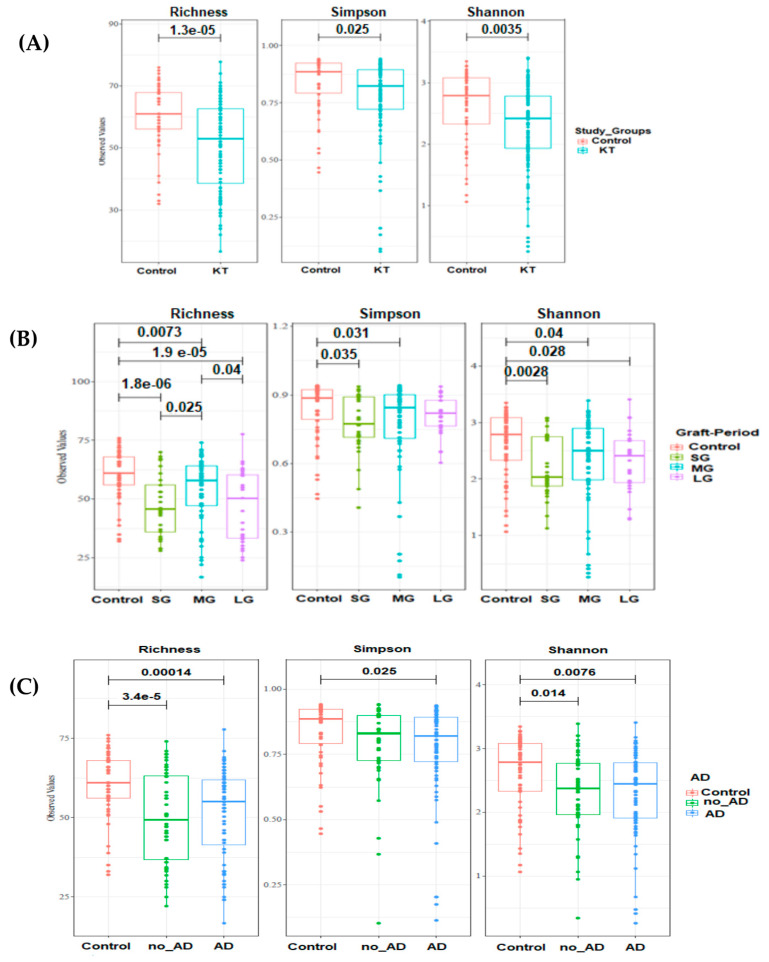
Boxplots of OTU’s richness, Simpson and Shannon diversity indexes distinguishing between kidney transplant (KT group) and control group. Statistical analysis was performed with Wilcoxon rank-sum test. (**A**) Alpha diversity indexes within control (n = 18) and kidney groups (n = 40). (**B**) Alpha diversity analysis among kidney transplant recipients over time: short period group: <1 year (SG; n = 11), medium-length period group: 1–10 years (MG; n = 20), and long period group: over 20 years (LG; n = 9). (**C**) Richness and diversity indexes within patients with and with no associated diseases (AD) groups (n = 24 and n = 16 respectively). Study groups are statistically different (*p*-value < 0.05).

**Figure 2:**
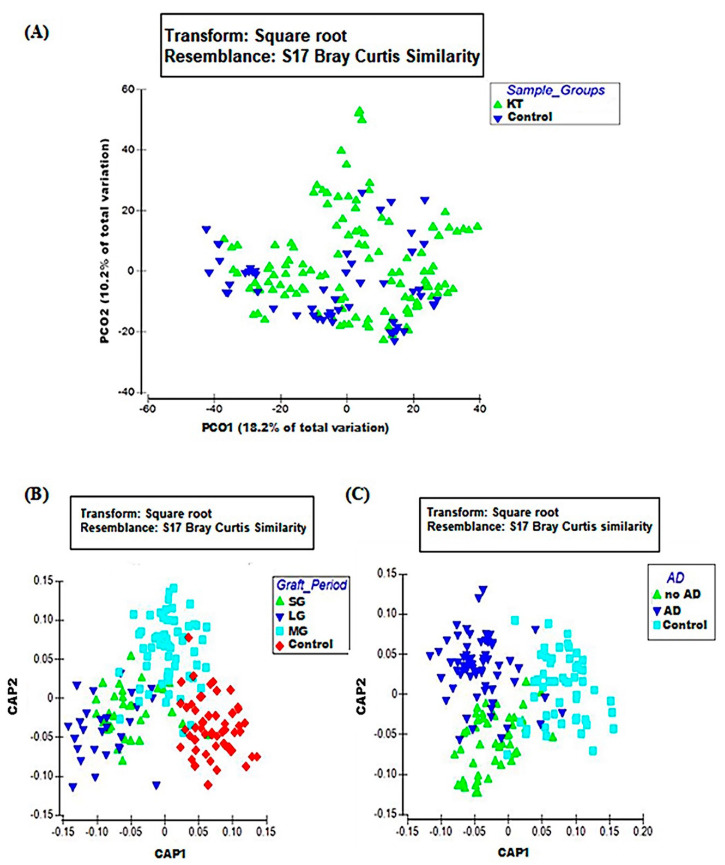
Beta diversity analysis of study groups; (**A**) PCO plot of KT group (green triangles; n = 40) and the control group (blue triangles; n = 18). (**B**) CAP plot of period factor subgroups; short (green triangles; n = 11), long (blue triangles; n = 9), medium (blue squares; n = 20), and the control group (red diamonds). (**C**) CAP plot of associated diseases (AD) factor subgroups; no AD (green triangles; n = 16), AD (blue triangles; n = 24), control (blue squares). Data matrix was pre-treated by square root transformation followed by resemblance analysis based on Bray–Curtis similarity calculation (*p* = 0.001).

The microbial community in kidney transplant (KT) patients was significantly different compared to healthy subjects. Alpha diversity (within-sample diversity) analysis showed significant difference in both microbial diversity and richness between study groups. In fact, the median Shannon diversity index was lower in the KT fecal specimens than in the control group (2.3 vs. 2.7 respectively, *p* = 0.0035, Wilcoxon rank-sum test) (Figure 1 and Supplementary Table S1). Meanwhile, a higher median richness index was noticeable within the control group than KT group (61 vs. 53 respectively, *p* = 1.3 × 10^−5^, Wilcoxon rank-sum test) (Figure 1 and Supplementary Table S1). Principal coordinates analysis (PCoA) was performed using unweighted Unifrac metric of fecal microbiota among all samples. The KT group was found to be significantly different from the control group (*p* = 0.001), as shown by PCoA analysis (Figure 2A). 

At the phylum level, Bacteroidetes and Firmicutes were the most dominant members of the gut microbiota present in the fecal specimens in control and KT patients (mean ± SE; 57.57% ± 1.83% vs. 50.96% ± 2.14% and 33.83% ± 1.57% vs. 33.63% ± 2.04% respectively). Additionally, bacterial profiles showed the presence of Actinobacteria and Verrucomicrobia phyla with respectively higher relative abundances among the control individuals (mean ± SE; 1.87% ± 0.34%; 1.51% ± 0.36%) than in the patient group (0.77% ± 0.12%; 0.41% ± 0.16%). Interestingly, Proteobacteria were found to be three times more abundant within the KT group (13.18% ± 1.40% vs. 4.94% ± 0.69%) (Supplementary Figure S1A). 

**Figure 3:**
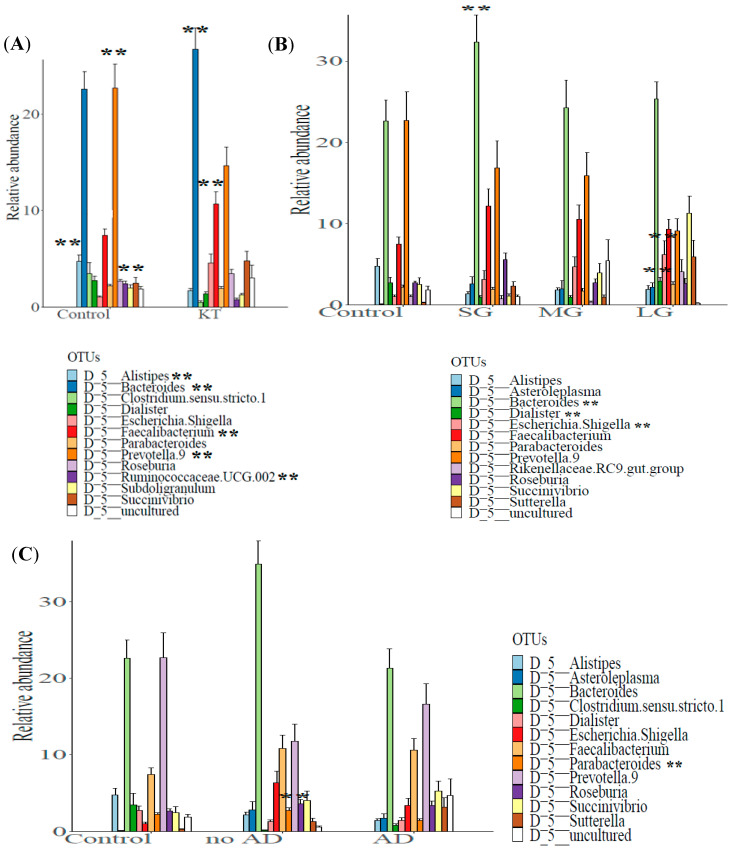
Comparison of the mean relative abundance of bacterial operational taxonomic units (OTUs) at genus (taxonomic level L6) between (**A**) control group (n = 18) vs. kidney transplant recipients (n = 40), (**B**) control subjects and patients after short (n = 11), medium (n = 20), and long post graft-period (n = 9), and (**C**) control subjects; patients suffering and non-suffering from associated diseases (n = 24 and n = 16 respectively). **: high statistical significance.

Evaluation of the most common taxa (>1% mean relative abundance among all fecal specimens) was performed at the genus level. The relative abundance of *Ruminococcaceae.UCG.002*, *Clostridium sensu strico 1*, *Subdoligranulum*, *Dialister*, *Parabacteroides*, *Alistipes*, and *Prevotella 9* was significantly lower and the relative abundance of *Escherichia/Shigella*, *Roseburia*, *Succinivibrio*, *Faecalibacterium*, and *Bacteroides* was higher in the KT fecal specimens than in the fecal specimens from the control group (Supplementary Table S2). A bar chart of the 12 most abundant genera is shown, with 7 genera being lower in the KT fecal specimens and 5 genera being higher in the KT fecal specimens compared to the fecal specimens from the control group (Figure 3A).

Serial group comparison analysis enabled us to identify five genera that were significantly different between kidney transplant group and control group. These genera were *Faecalibacterium*, other members of the Ruminococcaceae family, as well as *Alistipes, Bacteroides*, and *Prevotella 9* (Adj-*p*-values: *p* = 10^−4^; 0; 0.0057; 0.0047; *p* = 0.007 respectively, Wilcoxon rank-sum test) (Supplementary Figure S2). 

### 3.3. Bacterial Networking in the Gut 

**Figure 4:**
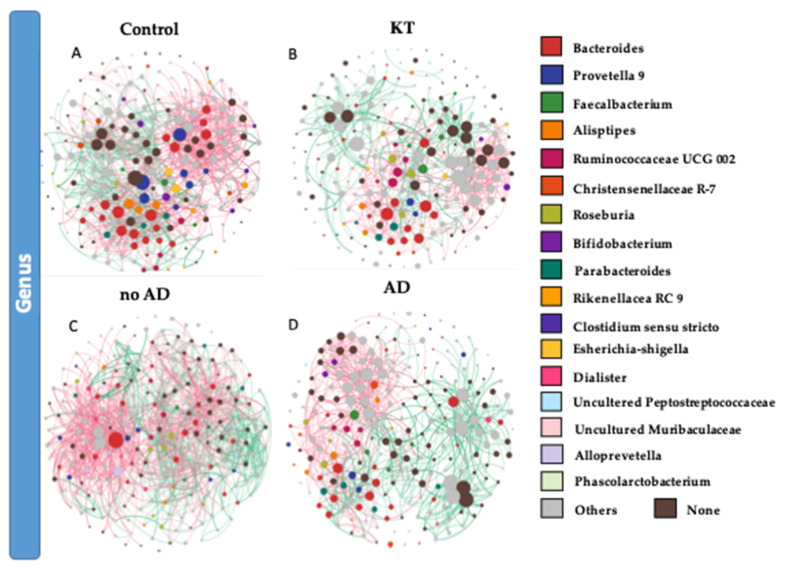

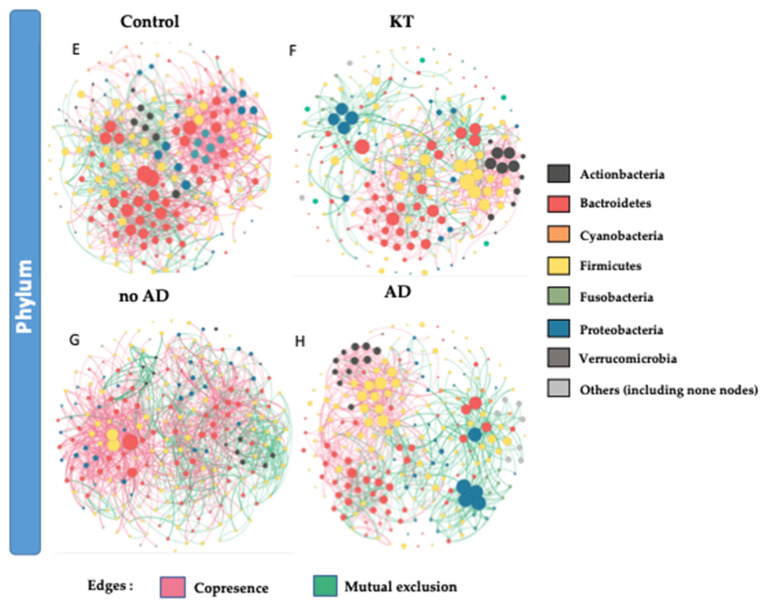
(**A**,**B**,**E**,**F**) Significant co-occurrence (red edges) and mutual exclusion (green edges) network analysis. Interactions among OTUs in the control group (n = 18) and patient group (n = 40). (**C**,**D**,**G**,**H**) Interaction among OTUs in no AD patients group (n = 16) and AD patient group (n = 24). For each group, the nodes correspond to the present OTUs colored according to phylum affiliation. The size of the nodes is proportional to their degree of connection (the number of edges associated to the node).

To identify potential interactions and niche-sharing among bacterial partners, we constructed inter-kingdom co-occurrence and mutual exclusion networks related to the KT patients and healthy subjects. The topological properties are shown in Supplementary Table S5. The two networks were visualized at both the genus and phylum levels and found to exhibit significantly different network structures (Figure 4A,B,G,H). Although, the number of nodes in kidney transplant recipients and control subjects was not variable (227 and 226 respectively), the number of interactions (edges) was found to be higher within the control group than in the KT group, namely 1417 and 894, respectively. Network analysis also indicated a higher clustering coefficient within the control group (0.245 vs. 0.177). This indicates that the network structures of gut communities are significantly altered in the kidney transplant group (Figure 4B,F), showing lower complexity and connectivity than those of healthy subjects (Supplementary Table S5, Figure 4A,G). Since the size of the nodes is proportional to their degree of connection, network visualization suggests that Bacteroidetes and Firmicutes possess a higher degree of interaction among all bacterial phyla in the gut of both groups, along with Proteobacteria and Actinobacteria in the KT group (Figure 4A,B,E,F and Supplementary Figure S5). A closer look at the profile of hub bacterial genera (genera showing the highest number of significant positive and/or negative correlations with other members of the community) revealed that in the gut microbiota of control individuals, the genera *Bacteroides* and *Prevotella 9* had the highest number of connections (mostly mutual exclusion) with the rest of the community, while in the microbiota of kidney transplanted group, the genera *Bacteroides*, *Prevotella 2*, and *Succinivibrio* had the highest number of connections (mutual exclusion relationships) within the community (Supplementary Figure S5A,B). 

### 3.4. Analysis of Factors Influencing Gut Bacterial Structure within Kidney Grafted Subjects 

#### 3.4.1. Graft Period 

Alpha diversity analysis showed a significant difference in both microbial diversity and richness between control and the three patient subgroups (Figure 1B). The median Shannon diversity index was significantly lower in the patient subgroups when compared to the control group (median Shannon index = 2 in “SG”, 2.52 in “MG”, and 2.46 in “LG” vs. 2.75 within the control group, *p* = 0.0028, *p* = 0.04, *p* = 0.028, respectively; Wilcoxon rank-sum test). However, there was no significant difference in Shannon diversity index between the patient’s subgroups when compared pairwise (*p* > 0.05). Similarly, according to the median richness index, a significant difference was found between the control and all three patient subgroups (*p* = 1.8 × 10^−6^, *p* = 0.0073, *p* = 1.9 × 10^−5^; Wilcoxon rank-sum test). Moreover, a significant difference was also found between “SG” and “MG” (*p* = 0.025), as well as between “MG” and “LG” (*p* = 0.04) (Figure 1B). 

Beta diversity performing canonical analysis of principal coordinates (CAP), calculated the variation of bacterial profiles between KT and control groups. Contrarily to PCoA, CAP visualization showed a clear separation between the control subjects and KT patients over time (Figure 2B). Relative abundances at the phylum level indicated differences in the bacterial structure between the control group and the over time subgroups. Bacteroidetes and Firmicutes phyla were notably lower among patients after a long post-graft period compared to healthy controls (45.15% ± 2.39% vs. 57.57 ± 2.59 and 26.50 ± 1.94 vs. 33.83 ± 2.23 respectively). Proteobacteria also showed significantly higher relative abundances over time; it represented only 4.49% ± 0.98% within the control subjects and increased from 7.07% ± 1.12% within the “SG” group to 10.66% ± 1.45% after 1 year post-graft “MG” to reach 26.28% ± 2.76% within patients after a long post-renal graft period “LG”(over 10 years) (Supplementary Figure S1B). 

The analysis of relative abundances at the genus level showed marked differences in the bacterial profiles between the control and the over time patients’ subgroups. The relative abundance of *Asteroleplasma*, *Roseburia*, *Faecalibacterium*, and *Bacteroides* was notably higher in fecal specimens of patients after the short transplant period (“SG”), while the relative abundance of *Rikenellaceae RC9 gut group*, *Dialister*, *Parabacteroides*, *Sutterella*, *Escherichia/Shigella*, and *Succinivibrio* was higher in patients after the long period of renal graft (“LG”). Among the twelve more abundant bacteria at the genus level described in Figure 3B, the genera *Alistipes* and *Prevotella 9* appeared to be more abundant in the control group compared to the KT subgroups over time (Supplementary Table S3 and Figure 3B). Serial group comparison analysis was performed to detect significant differences in taxa relative abundances. We found that the *Dialister* genus was significantly more abundant in “LG” compared to “SG” and “MG” (2.90 ± 0.47 vs. 0.95 ± 0.21 and 0.94 ± 0.21 Adj-*p*-value = 0 and Adj-*p*-value = 0.0084, respectively; Wilcoxon rank sum test). The *Sutterella* genus was also found to be more abundant after the long graft period compared with SG and MG, but this difference was not statistically significant (5.91% ± 2.05% vs. 2.28% ± 0.59% and 0.93% ± 0.31%; *p* > 0.05); in control individuals, it only represented 0.26% ± 0.08% of the fecal bacterial genera. Additionally, a significantly higher relative abundance of *Escherichia/Shigella* and *Bacteroides* was observed between “SG” and “MG” (3.11 ± 1.08 vs. 4.67 ± 1.22 Adj-*p*-values = 0.025; 32.37 ± 3.30 vs. 24.29 ± 3.36 Adj-*p*-values = 0.04, respectively) (Supplementary Figure S3).

#### 3.4.2. Associated Diseases

Alpha diversity and beta-diversity analysis were performed to investigate differences in whole gut microbial diversity in patients with and without associated diseases (ADs) after kidney transplantation compared to healthy controls (Figure 1C and Figure 2C). 

Relative abundances analysis showed that the phylum Bacteroidetes had a lower relative abundance in the patient groups with associated diseases (AD subgroup) compared to patients not suffering from other diseases and to healthy controls (mean relative abundance ± SE; “Control” 48.48% ± 2.77% vs. “AD” 57.57% ± 2.37%; “Control” vs. “No AD” 54.68% ± 2.74%; “AD” vs. “No AD”). 

The evaluation of the most common taxa at the genus level showed a higher relative abundance of *Asteroleplasma*, *Parabacteroides*, *Alistipes*, *Roseburia*, *Escherichia.Shigella*, *Faecalibacterium*, and *Bacteroides* in the samples from the no AD subgroup, including individuals not suffering from associated diseases. While the genera of *Sutterella* and *Succinivibrio* were highly abundant in the subgroup of patients with post graft complications (AD), *Clostridium sensu stricto 1*, *Dialister*, *Alistipes*, and *Prevotella 9* were more abundant among control individuals (Supplementary Table S4 and Figure 3C).

Serial group comparison analysis was performed in order to detect significant differences in taxa composition and abundances between mainly AD and no AD groups. Contrarily to the relative abundance results, we only identified the *Parabacteroides* genus that was significantly different between the two patient subgroups compared pairwise (Adj-*p*-value = 3 × 10^−3^) and to the control group. (Adj-*p*-value = 3 × 10^−4^). Serial group comparison was calculated using the Wilcoxon rank sum test (Supplementary Figure S4). 

Bacterial co-occurrence/mutual exclusion networks indicated marked differences between OTUs found in no AD patients and AD patients (Figure 4C–H). The topological properties are shown in Supplementary Table S5. The number of nodes in kidney transplant recipients with associated diseases and not suffering from ADs was not variable (234 and 230, respectively), but the number of interactions (edges) was lower in the AD group than the healthier patient group (1130 and 1704, respectively). Network topology analysis showed a higher clustering coefficient within the no AD group (0.282 vs. 0.234 within AD group). This indicates lower complexity and connectivity in patients suffering from ADs than those with no ADs (Supplementary Table S5, Figure 4C–H). The network analysis profile of genera showing the highest number of significant positive and/or negative correlations with other members of the community revealed that in the gut microbiota of the no AD group, the genera *Bacteroides* and *Alloprevotella* and the *Ruminococcus gnavus* group had the highest number of connections (mostly mutual exclusion) with the rest of the community, while in the microbiota of the AD group, the genera *Bacteroides*, *Prevotella 2*, and *Sutterella* had the highest number of connections (mutual exclusion relationships) within the community (Supplementary Figure S5C,D).

## 4. Discussion

The current study offers new insights into the gut dysbiosis that occurs within stable transplant patients. Our investigation of the gut microbiota was performed by PCR amplification of the 16S rRNA V3–V4 variable region and deep sequencing using an Illumina MiSeq platform. This method helped to identify significant alterations in the gut microbial composition that may be associated with graft period and associated diseases. Thus, there may be some possible limitations in this study. Mainly, the sample size could be the reason why it was difficult to identify significant relationships between the age factor and the kidney transplantation.

### 4.1. Decrease in Gut Microbiota Richness and Diversity in Kidney Allograft Patients

Recent studies have evaluated the microbial community in fecal samples of kidney transplant recipients and revealed a significant shift in their post-transplant microbiome composition. Fricke et al. reported that significant shifts in microbiota composition could be identified one month after transplantation, and that the pre-transplant microbiota differences can be connected with post-transplant events (e.g., infectious) [26]. Herein, we studied the differences in gut microbial profiles between stable kidney-transplanted patients at different post-transplant time intervals (from 3 months to 22 years post-graft) and healthy controls. Alpha diversity analysis was conducted to investigate within-group diversity. Richness index revealed significantly lower bacterial richness after renal graft when compared to the control group. This result is directly in line with previous findings in the context of chronic kidney diseases and kidney graft. In a recent study, FengXia Li et al. (2019) showed a significant reduction in the overall richness of the gut microbiota in Chinese patients with chronic kidney disease (CKD) compared to healthy control subjects [27]. Consistent with that, another study investigating gut bacterial composition of KT, CKD, and healthy control groups revealed that kidney transplanted samples had the lowest richness, followed by CKD, and the highest microbial richness occurred in the controls [28]. This result provides convincing evidence that the gut microbiota is clinically altered after kidney transplantation. However, increased microbiota richness shortly after organ graft was found to be correlated with immunosuppressive therapy (the corticosteroid prednisone) when compared to pretransplant microbiota [29]. 

In the present study, the median Shannon diversity index was lower in the KT group compared to the control group. In line with our finding, several studies showed that low diversity was associated with renal graft [30]. Of note, studies in human and mice reported that a reduction in the diversity of microbiota is associated with post-transplant infection and acute rejection in liver graft recipients [31].

The evaluation of the gut microbiota’s structure in kidney transplant recipients and in CKD patients will pave the way to new tools to restore a microbial balance that is more conducive to health and will help identify new non-invasive approaches to diagnose and treat renal complications and help confer a better quality of life for this population.

### 4.2. Graft Period Impacts Gut Microbial Structure

Some clinical trials have been performed to characterize the gut microbiota after kidney transplantation [9,11,27,29]. These short-term studies were designed to assess for correlations with endpoints such as acute rejection, with characterization only at the taxa level through 16S rRNA gene sequencing, though the long-term variation of gut microbiota remains an unanswered and challenging question, as most of the renal transplants still undergo rejection even after more than a decade or two of their transplantation [32]. In this pilot study, we characterized the gut microbiota at the phylum and genus level and at different time intervals after kidney transplantation. Herein, we aim to study the possible impact of the immunosuppressive therapy length on both microbial structure and networking.

Taur et al. (2012) previously reported that immunosuppressive therapies (e.g., tacrolimus and mycophenolate mofetil) in mice modify innate antimicrobial defenses and disturb gut microbiota [33]. Furthermore, human studies of the gut microbiota in RT recipients and CKD patients characterized the short-term dysbiosis. At the phylum level, they showed an increased abundance of Proteobacteria and Bacteroidetes and a decreased abundance in Firmicutes after 1-month post-transplant compared to healthy controls. In the present study, the metagenomic analysis suggests that at the phylum level, Bacteroidetes and Firmicutes were the most abundant bacterial taxa in both the post-transplant group and healthy subjects. A similar pattern of results was characterized by the Human Microbiome Consortium (2012) for healthy individuals [34] and by Mahmoodpoor et al. (2017) in the context of kidney pathogenesis [35]. Of note, in the gut microbiota, the relative abundance of Bacteroidetes and Firmicutes phyla is >90% [36]. Studies in mice and humans found that an increased ratio of Firmicutes/Bacteroidetes was noticed in the gut microbiota in populations with obesity [37], hypertension [38] and diabetes [39]. 

We also observed significant differences in the composition of gut microbiota over time after kidney transplant. Our data showed a three-fold increase in the relative abundance of Proteobacteria within the KT group, reaching 26% after a long graft period. A study by Lee et al. (2014), reported a significant increase in the mean fecal abundance of Proteobacteria within one month after kidney transplant, in line with our results [9]. As for the long-term proliferation of this phylum, little is known. Proteobacteria are currently the largest phylum within the bacteria domain. This phylum is often overrepresented in several intestinal and extraintestinal diseases, mostly with inflammatory phenotypes. Thus, it is considered to be a common factor in human diseases [40]. In the present study, we can suggest that the Proteobacteria phylum propagates over time continuously after kidney transplantation, taking advantage of the absence of human defenses caused by the immunosuppressive therapy. Our evaluation of the gut phyla showed significant lower abundances of Actinobacteria and Verrucomicrobia shortly after renal graft compared to the control group. This finding opposes the results found by Li et al. (2019), who reported an increase in the abundance of these phyla after kidney graft [27]. Notably, Actinobacteria are Gram-positive bacteria that constitute one of the five largest bacterial phyla and produce antibiotic, anticancer, anthelmintic, and antifungal compounds that are important in biotechnology, medicine, and/or agriculture [41]. However, the roles of both Actinobacteria and Verrucomicrobia in CKD are not yet clear. 

At the genus level, we found that *Bacteroides* and *Faecalibacterium* were highly abundant after kidney graft compared to healthy controls. Bacteria in the *Faecalibacterium* genus degrade glucose, fructose, fructooligosaccharides, and complex molecules such as pectin [42]. This might explain weight loss that occurs in this population shortly after the surgery. However, we found decreased abundances of *Faecalibacterium* and *Prevotella 9* in KT recipients after long graft period. Most of these microorganisms are anaerobic or facultative anaerobic probiotics and produce short-chain fatty acids (SCFAs). Emerging evidence shows that SCFAs seem to be the link that connects gut microbiota and host homeostasis [43]. In fact, an association between SCFAs and renal injury was recently revealed. SCFAs were found to possibly interfere with the progression of CKD in several ways, including controlling blood pressure by inducing renin secretion and the development of subtypes of renal cells [44]. In line with the present results, Jiang et al. (2017) found that butyrate-producing bacteria, including *Faecalibacterium*, *Clostridium*, *Coprococcus*, and *Prevotella*, were reduced in an end-stage renal disease (ESRD) group [45]. Another research team reported that treatment with acetate-producing bacteria helped increase acetate levels and ameliorate acute renal injury [46]. This evidence suggests that SCFAs could represent not only metabolic biomarkers in the context of kidney disease, but they may also act as new therapeutic targets for prevention of renal injury.

In addition, the relative abundances of *Escherichia coli*, *Sutterella*, and *Dialister* showed a significant increase after the renal graft. This increase was found to be gradual over time for *Escherichia.Shigella*, namely from 3.11% within “SG” to 4.67% after 1-year post-graft and to 6.16% within patients in the “LG”. Furthermore, in the study by Jiang et al. (2017), it was shown that the genera *Escherichia.Shigella* were enriched in ESRD patients after six months of kidney graft, which is consistent with our results [45]. *E. coli* is the most frequent bacterial pathogen that causes pneumonia [47], urinary tract infection [48], and necrotizing fasciitis [49] after renal transplantation. *Sutterella* species have been frequently associated with human diseases such as autism, Down’s syndrome, inflammatory bowel disease (IBD) [50], and kidney stone disease [51]. Its pathogenicity is caused by its intestinal epithelial cells’ adhesion and mild pro-inflammatory capacities in the human gastrointestinal tract [52]. As for *Dialister*, this is a non-fermentative, anaerobic, Gram-negative rod that has pathogenic potential in dental root canals and various body sites, including the lung and brain [53]. However, the role of *Dialister* in kidney transplantation is not yet clear. Mainly, these results show a shift in the level of colonization of some bacterial phyla over time, such us Firmicutes, Bacteroidetes, and Actinobacteria after a short period of transplantation, and Proteobacteria after a long time. It also reveals that at some point after surgery, new genera may appear, such as *Dialister* and *Sutterella*. All these changes could have a clinical impact on the health of patients after kidney transplantation.

### 4.3. Post-Transplant Complications Related to Dysbiosis

Several data support that the dysbiosis in bacterial structure and diversity are even more significant when post-operative complications occur [26,27]. In the present study, beta diversity analysis showed that patients with associated diseases such as high blood pressure, type II diabetes, obesity, and dyslipidemia (n = 24) had significantly different microbial patterns than renal graft recipients with no ADs (*p* = 0.008). Interestingly, serial group comparison by running a Kruskal–Wallis rank sum test along with a Wilcoxon rank sum showed a lower abundance of *Parabacteroides* within recipients suffering from associated diseases (AD group) when compared to patients with no AD. Species of *Bacteroides* and *Parabacteroides* represent opportunistic pathogens in infectious diseases, and they are able to develop antimicrobial drug resistance [54]. Consistent with our findings, metagenomic studies (in which bulk microbiome DNA was sequenced) have noted associations between certain species of *Parabacteroides* and diabetes [35,55]. Furthermore, gut microbiota was associated with metabolic diseases, mainly overweight. In a recent study, Chiu et al. (2014) found a positive association between *Parabacteroides distasonis* and obesity [56].

Dysbiosis can also be a result of the maintained immunosuppressive therapy after kidney graft. For instance, it is reported that immunosuppression-driven dysbiosis causes the overgrowth of *E. coli* and opportunistic pathogens [57]. Moreover, studies on rat models have shown, at the metagenomic level, that immunosuppression leads to a more catabolic microbial profile with an increase of genes involved in sucrose degradation, similar to diabetes, which may influence the development of diabetes after solid organ transplantation. Conversely, the control rats had a greater abundance of anabolic processes and genes involved in starch degradation. The same team investigated the diabetogenic effect of immunosuppression within humans and found that significantly dysregulated genes in the context of immunosuppression are implicated in insulin signaling and secretion [54]. Altogether, pieces of evidence explain the occurrence of type II diabetes, obesity, and high blood pressure after stable kidney allograft. 

Other complications related to post graft dysbiosis are shown through the network analysis and bacterial co-occurrence visualization among members of the gut microbiota. Our interaction network analysis revealed that within the microbiota of stool contents, the occurrence of lifestyle diseases resulted in an increased number of positive interactions of Actinobacteria and Firmicutes genera with other members of the community. Moreover, the present findings indicated a higher inhibition rate of the Proteobacteria phylum over the rest of the community members. Mainly, *Succinivibrio* and *Sutterella* genera had a higher number of mutual exclusion interactions in kidney transplant recipients suffering from lifestyle diseases than those who were more stable. The increased abundance of the *Sutterella* genus has been observed to be involved in the pathogenesis of autism [58], while the *Succinivibrio* genus was described to cause bacteremia [59]. The potential effect of these bacterial genera on the gut community following kidney transplantation remains unknown. Notably, the Proteobacteria phylum is comprised of a broad range of Gram-negative bacteria and includes many known infectious pathogens, including *Escherichia coli* [9]. Moreover, members of Proteobacteria often have increases in type II diabetes, obesity, and non-alcoholic fatty liver disease [40], as well as in organ transplantation [60]. An increase in Enterobacteriaceae is often thought to signal a proinflammatory state and is associated with poor health status [60]. These data suggest that an increase in Proteobacteria after transplantation could potentially contribute to the high rate of infectious complications that lead to the appearance of lifestyle diseases over time. The network patterns in the no AD group reveal a possible distinction between the real impact of kidney transplantation and the combined impact of kidney graft and lifestyle diseases found in the AD group.

We can hypothesize that the differences in the number of OTUs as well as the members and potential of interactions between the AD patient and the no AD patient could represent specific markers that could enable us to distinguish whether the dysbiosis is due to the kidney graft itself or it is associated to different pathogenesis mechanisms like metabolic diseases.

Altogether, these reports offer a specific connotation between alterations in gut microbiota and the immune response that determines the allograft outcome in the kidney [30,33]. Moreover, post-transplant dysbiosis and complications are related to poor clinical allograft outcome and increased morbidity and mortality of graft recipients [35].

## 5. Conclusions

The differential gut microbiota has the potential to guide non-invasive diagnosis and targeted interventions. Given that many factors are associated with the diagnosis of gut microbiota, the biomarker is not a single bacterium but a set of bacteria. The present study found associated phylotypes by analyzing data from the high-throughput sequencing of the16S rRNA gene, but the reliability of these bacteria as biomarkers needs to be explored further. Hence, large-scale prospective studies with large sample panels, especially pre-transplant samples acting as their own controls, should be conducted with the aim of developing more reliable microbiome biomarkers.

Our study shows specific kidney transplant-related effects of the fecal microbiome on graft stability and patient’s health status when compared to healthy subjects. Such findings suggest that the analysis of the gut microbial community could represent a new tool to better evaluate the effects of drugs currently employed in organ transplantations. Our results show that alterations in the composition of gut microbiota are significantly correlated with the clinical conditions of renal transplant recipients, and future studies of these correlations may provide evidence for predicting the clinical outcomes of RT recipients. A better understanding of the relationship between gut microbiota and the complications related to allogeneic transplantation may convert gut microbiota into a diagnostic and therapeutic target in the future. 

## Data Availability

All sequence files are available from the SRA NCBI database using the following information: Bioproject; Accession: PRJNA623593, URL for all samples: https://www.ncbi.nlm.nih.gov/sra/PRJNA623593.

## Supplementary Materials

The following are available online at https://www.mdpi.com/2076-2607/8/11/1724/s1. Figure S1: Comparison of the mean relative abundance of bacterial operational taxonomic units (OTUs) at phylum (taxonomic level L2) between study groups; Figure S2: Serial group comparison analysis. Box plot of significantly different OTUs between control and KT individuals (Wilcoxon rank sum test); Figure S3. Serial group comparison analysis. Box plot of significantly different OTU between patient’s groups over time and healthy individuals; Figure S4. Serial group comparison analysis. Box plot of significantly different OTU between kidney graft recipients suffering and not suffering from lifestyle diseases; Figure S5. Total number of positive (Co-presence/Co-occurrence) and negative (Mutual exclusion) relationships between nodes at Genus level within study groups; Table S1: Richness and diversity estimation of the 16S *rRNA* gene libraries of kidney transplanted patients through the amplicon sequence analysis; Table S2. Mean relative abundances of the twelve most abundant bacteria at genus level present in the faecal specimens of 40 kidney-transplant patients and 18 control subjects; Table S3. Mean relative abundances of the twelve most abundant bacteria at genus level present in faecal specimens of patients receiving a kidney graft over time and of healthy individuals; Table S4. Mean relative abundances of the twelve most abundant bacteria at genus level present in faecal specimens of kidney graft recipients suffering or not from associated diseases; Table S5. Topological properties of networks in fecal communities of healthy and kidney transplant samples.
