# Supplementary material for: Impact of the Post-Transplant Period and Lifestyle Diseases on Human Gut Microbiota in Kidney Graft Recipients
Source: Microorganisms. 2020 Nov 4;8(11):1724. doi: 10.3390/microorganisms8111724 (PMC7694191; doi:10.3390/microorganisms8111724)
Supplement: Supplementary file 1 [file microorganisms-08-01724-s001.zip › Figure S1.docx]

**Figure S1.** Comparison of the mean relative abundance of bacterial operational taxonomic units (OTUs) at phylum (taxonomic level L2) between study groups: **(A)** control group vs kidney transplant recipients, **(B)** control subjects and patients after short, medium and long post graft-period and **(C)** control subjects, patients suffering and non-suffering from associated diseases.
