# Supplementary material for: Impact of the Post-Transplant Period and Lifestyle Diseases on Human Gut Microbiota in Kidney Graft Recipients
Source: Microorganisms. 2020 Nov 4;8(11):1724. doi: 10.3390/microorganisms8111724 (PMC7694191; doi:10.3390/microorganisms8111724)
Supplement: Supplementary file 1 [file microorganisms-08-01724-s001.zip › Figure S3.docx]

**Figure S3.** Serial group comparison analysis. Box plot of significantly different OTU between patients groups over time and healthy individuals: patients receiving a kidney graft before short (1 year; n= 11); medium-length (2 to 10 years; n=20) and long (>10 years; n=9) periods and 18 control subjects (Wilcoxon rank sum test).
