# Supplementary material for: Impact of the Post-Transplant Period and Lifestyle Diseases on Human Gut Microbiota in Kidney Graft Recipients
Source: Microorganisms. 2020 Nov 4;8(11):1724. doi: 10.3390/microorganisms8111724 (PMC7694191; doi:10.3390/microorganisms8111724)
Supplement: Supplementary file 1 [file microorganisms-08-01724-s001.zip › Figure S4.docx]

**Figure S4.** Serial group comparison analysis. Box plot of significantly different OTU between kidney graft recipients suffering and not suffering from lifestyle diseases: (AD) (n= 24); and no AD (n=16) (Wilcoxon rank sum test).
