# Supplementary material for: Impact of the Post-Transplant Period and Lifestyle Diseases on Human Gut Microbiota in Kidney Graft Recipients
Source: Microorganisms. 2020 Nov 4;8(11):1724. doi: 10.3390/microorganisms8111724 (PMC7694191; doi:10.3390/microorganisms8111724)
Supplement: Supplementary file 1 [file microorganisms-08-01724-s001.zip › Figure S5.docx]

**
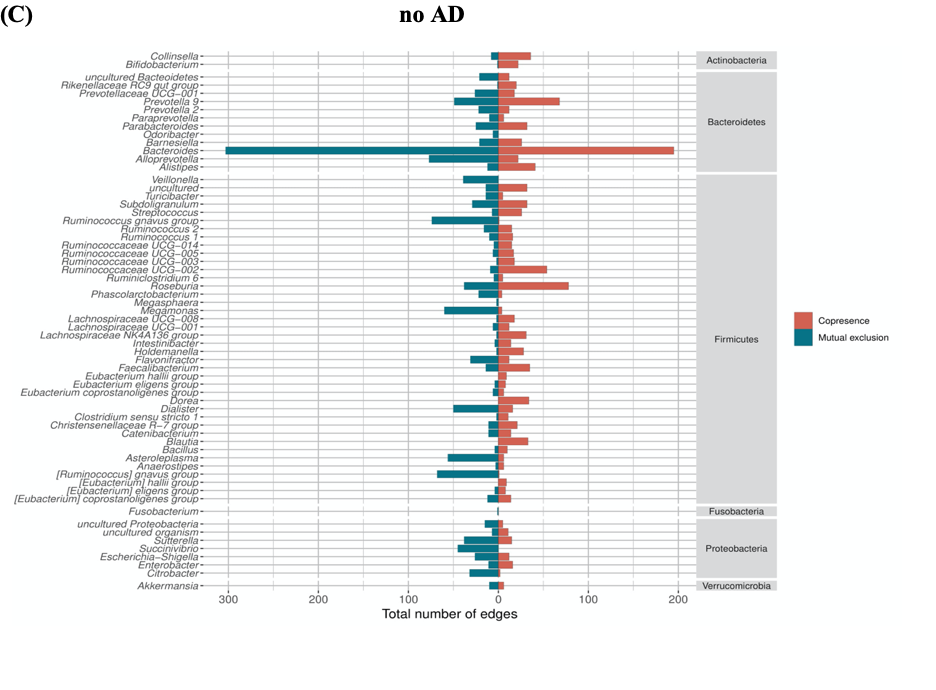
**

**
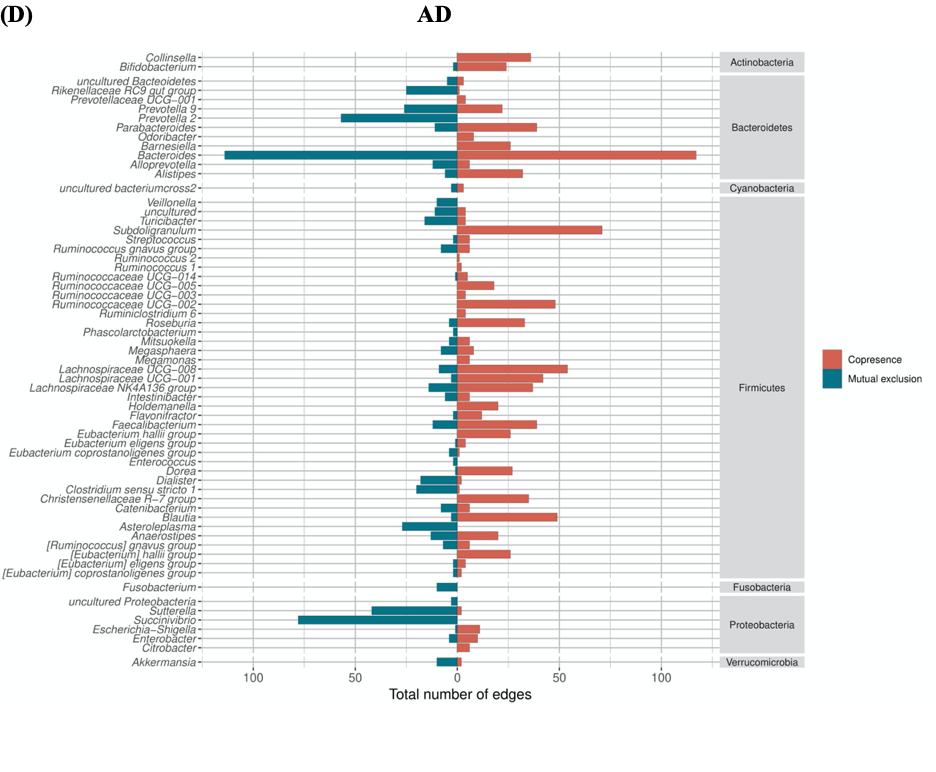
Figure S5.** Total number of positive (Co-presence/Co-occurrence) and negative (Mutual exclusion) relationships between nodes at Genus level within study groups (**A)** Control subjects**,** **(B)** Kidney transplant recipients**, (C)** Patients with no associated diseases**,** and (**D)** AD group.
