# Supplementary material for: Impact of the Post-Transplant Period and Lifestyle Diseases on Human Gut Microbiota in Kidney Graft Recipients
Source: Microorganisms. 2020 Nov 4;8(11):1724. doi: 10.3390/microorganisms8111724 (PMC7694191; doi:10.3390/microorganisms8111724)
Supplement: Supplementary file 1 [file microorganisms-08-01724-s001.zip › Nessrine et al. 2020 ALL Supplementary Documents.docx]

**SUPPLEMENTARY INFORMATION to manuscript**

**Impact of the post-transplant period and lifestyle diseases on human gut microbiota in kidney graft recipients.**

Nessrine Souai ^1,2^, Oumaima Zidi^1,2^, Amor Mosbah ^1^, Imen Kosai ^3^, Jameleddine El Manaa ^3^, Naima Bel Mokhtar ^4^, Elias Asimakis ^4^, Panagiota Stathopoulou ^4^, Ameur Cherif ^1^, George Tsiamis ^4^ and Soumaya Kouidhi^1^*

1 Laboratory of Biotechnology and Valorisation of Bio-GeoRessources, Higher Institute of Biotechnology of Sidi Thabet, BiotechPole of Sidi Thabet, University of Manouba, Ariana 2020, Tunisia. nessrine.souai@fst.utm.tn (N.S.); oumaima.zidi@hotmail.fr (O.Z.); amor.mosbah@gmail.com (A.M.); ameur.cherif@uma.tn (A.C.); soumayakouidhi@gmail.com (S.K).

2 Department of Biology, Faculty of Sciences of Tunis, University of Tunis El Manar, Farhat Hachad Universitary Campus, 1068 Rommana, Tunis, Tunisia; nessrine.souai@fst.utm.tn (N.S.); oumaima.zidi@hotmail.fr (O.Z.)

3 Unit of Organ Transplant Military Training Hospital, Mont Fleury-1008, Tunis, Tunisia; imenelkosai@gmail.com (I.K.); manaajamel@yahoo.fr (J.E.M.)

4 Laboratory of Systems Microbiology and Applied Genomics, Department of Environmental Engineering, University of Patras, 2 Seferi St, 30100 Agrinio, Greece; naima1503@gmail.com (N.B.M.); eliasasim@gmail.com (E.A.); panayotastathopoulou@gmail.com (P.S.); gtsiamis@upatras.gr (G.T.)

***** Correspondence: [soumayakouidhi@gmail.com](mailto:soumayakouidhi@gmail.com) (S.K); Tel: +216 95 694 135

**Table S1**. Richness and diversity estimation of the 16S *rRNA* gene libraries of kidney transplanted patients through the amplicon sequence analysis. (M: male, F: female), Asterisks indicates groups similarity calculated by Student t-test, *P*<.05. Significant differences are indicated by different letters.

|  | **Age** | **Gender** | **Number of Reads** | **OTU number** | **Richness** | **Simpson** | **Shannon** | **Pielous**  **evenness** |
| --- | --- | --- | --- | --- | --- | --- | --- | --- |
| **P1** | 21 | M | 6935 | 295.09±5.27 | 44.33±0.33^a^ | 0.9±0.002 ^a^ | 2.77±0.01 ^a^ | 0.73±0.004 ^a^ |
| **P2** | 53 | M | 13615 | 326.11±3.86 | 37.67±3.71^a^ | 0.77±0.02 ^b^ | 2.01±0.06 ^b^ | 0.56±0.03 ^b^ |
| **P3** | 35 | F | 3883 | 324.33±1.63 | 63±3.21^b^ | 0.92±0.01 ^b^ | 3.05±0.05 ^c^ | 0.74±0.01 ^a^ |
| **P4** | 47 | M | 3829 | 338.66±0.19 | 45.67±4.06^c^ | 0.67±0.01 ^c^ | 1.93±0.04 ^b^ | 0.51±0.01 ^b^ |
| **P5** | 67 | M | 1768 | 282.1±3.46 | 51.67±6.01^a^ | 0.9±0.01 ^a^ | 2.98±0.1 ^c^ | 0.76±0.006 ^a^ |
| **P6** | 37 | M | 13210 | 310.83±4.26 | 57.28±7.14^d^ | 0.86±0.009 ^a^ | 2.54±0.06 ^d^ | 0.63±0.04 ^b^ |
| **P7** | 37 | M | 17736 | 314.14±1.66 | 57.28±2.29^d^ | 0.89±0.003 ^a^ | 2.72±0.03 ^d^ | 0.67±0.01 ^b^ |
| **P8** | 66 | M | 12803 | 304.67±7.05 | 64.99±3.05^d^ | 0.92±0.01 ^b^ | 2.98±0.07 ^c^ | 0.71±0.02 ^b^ |
| **P9** | 48 | F | 13410 | 323.41±15.62 | 29.28±4.02^d^ | 0.3±0.1 ^d^ | 0.78±0.23 ^e^ | 0.23±0.06 ^c^ |
| **P10** | 18 | F | 17780 | 331.34±0.56 | 34.97±4.14^a^ | 0.79±0.01 ^b^ | 1.98±0.03 ^b^ | 0.56±0.02 ^b^ |
| **P11** | 29 | M | 15986 | 317.02±1.88 | 64.64±0.87^d^ | 0.82±0.01 ^b^ | 2.47±0.03 ^d^ | 0.59±0.01 ^b^ |
| **P12** | 58 | M | 26812 | 255.44±8.84 | 63.66±5.36^d^ | 0.93±0.01 ^b^ | 3.01±0.09 ^c^ | 0.73±0.01 ^a^ |
| **P13** | 33 | F | 17208 | 340.21±2.52 | 53.56±5.4^a^ | 0.74±0.02 ^c^ | 2.05±0.04 ^b^ | 0.52±0.01 ^b^ |
| **P14** | 56 | M | 15223 | 328.69±2.27 | 54.3±2.3^a^ | 0.77±0.01 ^b^ | 2.01±0.09 ^b^ | 0.5±0.03 ^b^ |
| **P15** | 39 | M | 13777 | 301.7±3.05 | 55.97±11.49^d^ | 0.93±0.01 ^b^ | 3.02±0.04 ^c^ | 0.77±0.06 ^a^ |
| **P16** | 57 | M | 16283 | 268.42±16.63 | 65.67±3.84 ^d^ | 0.85±0.08 ^a^ | 2.9±0.4 ^c^ | 0.69±0.09 ^b^ |
| **P17** | 53 | M | 1755 | 299.22±3.59 | 29.67±2.85 ^d^ | 0.75±0.07 ^c^ | 1.99±0.27 ^b^ | 0.6±0.1 ^b^ |
| **P18** | 51 | M | 10328 | 283.12±3.45 | 65±0.58 ^d^ | 0.92±0.01 ^b^ | 3.09±0.05 ^c^ | 0.74±0.01 ^a^ |
| **P19** | 46 | M | 12513 | 322.73±7.05 | 53.66±3.84 ^a^ | 0.83±0.07 ^b^ | 2.54±0.29 ^d^ | 0.64±0.06 ^b^ |
| **P20** | 36 | F | 7399 | 315.23±4.28 | 27.67±1.86 ^d^ | 0.69±0.04 ^c^ | 1.73±0.13 ^b^ | 0.52±0.03 ^b^ |
| **P21** | 60 | F | 12115 | 331.8±3.9 | 50.66±2.34 ^a^ | 0.56±0.12 ^b^ | 1.56±0.33 ^b^ | 0.4±0.08 ^b^ |
| **P22** | 17 | M | 9962 | 308.17±4.76 | 46.66±0.88 ^c^ | 0.85±0.03 ^a^ | 2.54±0.14 ^d^ | 0.66±0.03 ^b^ |
| **P23** | 26 | M | 14075 | 333.82±10.9 | 35.65±0.88 ^a^ | 0.73±0.02 ^c^ | 1.78±0.11 ^b^ | 0.5±0.03 ^b^ |
| **P24** | 42 | F | 16675 | 323.93±12.15 | 49.58±9.82 ^a^ | 0.81±0.04 ^b^ | 2.3±0.24 ^b^ | 0.59±0.03 ^b^ |
| **P25** | 43 | F | 22664 | 333.07±2.2 | 60.12±2.16 ^d^ | 0.81±0.01 ^b^ | 2.35±0.1 ^b^ | 0.57±0.02 ^b^ |
| **P26** | 42 | F | 14568 | 308.01±4.79 | 59.33±1.76 ^d^ | 0.81±0.02 ^b^ | 2.45±0.06 ^d^ | 0.6±0.02 ^b^ |
| **P27** | 50 | F | 14450 | 325.05±20.47 | 51.33±8.84 ^a^ | 0.68±0.1 ^c^ | 2.06±0.36 ^b^ | 0.52±0.07 ^b^ |
| **P28** | 41 | M | 22288 | 320.42±14.03 | 64.22±4.21 ^d^ | 0.8±0.11 ^b^ | 2.55±0.39 ^d^ | 0.61±0.09 ^b^ |
| **P29** | 27 | M | 21007 | 312.23±6.97 | 66.63±2.34 ^d^ | 0.7±0.02 ^c^ | 2.17±0.09 ^b^ | 0.52±0.02 ^b^ |
| **P30** | 61 | M | 15217 | 231.8±2.54 | 56.33±1.86 ^d^ | 0.83±0.03 ^b^ | 2.5±0.1 ^d^ | 0.62±0.02 ^b^ |
| **P31** | 43 | F | 29035 | 345.23±5.3 | 34.98±1.08 ^a^ | 0.8±0.02 ^b^ | 1.94±0.09 ^b^ | 0.54±0.03 ^b^ |
| **P32** | 37 | M | 19795 | 339.4±12.54 | 61.33±3.59 ^d^ | 0.58±0.2 ^b^ | 1.85±0.65 ^b^ | 0.45±0.16 ^b^ |
| **P33** | 30 | M | 15420 | 327.39±4.58 | 29.66±1.2 ^d^ | 0.71±0.01 ^c^ | 1.74±0.03 ^b^ | 0.51±0 ^b^ |
| **P34** | 50 | M | 21069 | 310.85±2.38 | 60.97±2.06 ^d^ | 0.78±0.01 ^b^ | 2.43±0.06 ^b^ | 0.59±0.01 ^b^ |
| **P35** | 41 | M | 10042 | 338.47±5.86 | 28.27±1.97 ^d^ | 0.73±0.08 ^c^ | 1.76±0.46 ^d^ | 0.53±0.15 ^b^ |
| **P36** | 30 | M | 12602 | 318.01±4.05 | 49.33±3.29 ^a^ | 0.85±0.01 ^a^ | 2.55±0.09 ^d^ | 0.66±0.02 ^b^ |
| **P37** | 39 | M | 47005 | 308.19±2.32 | 49.66±1.22 ^a^ | 0.86±0.02 ^a^ | 2.53±0.15 ^c^ | 0.64±0.04 ^b^ |
| **P38** | 47 | M | 42892 | 325.16±7.68 | 62.94±7.99 ^b^ | 0.92±0.01 ^b^ | 2.99±0.21 ^c^ | 0.72±0.03 ^b^ |
| **P39** | 42 | F | 72013 | 334.21±4.72 | 64.6±2.58 ^d^ | 0.9±0.01 ^a^ | 2.87±0.12 ^b^ | 0.69±0.02 ^b^ |
| **P40** | 44 | M | 21589 | 371.64±0.38 | 24.68±4.55 ^d^ | 0.16±0.03 ^d^ | 0.38±0.06 ^b^ | 0.12±0.01 ^b^ |
| **C1** | 24 | F | 35517 | 278.76±5.01 | 66.92±3.6 ^d^ | 0.72±0.06 ^c^ | 2.18±0.34 ^d^ | 0.52±0.08 ^b^ |
| **C2** | 24 | F | 22288 | 299.5±1.6 | 60.58±6.32 ^d^ | 0.84±0.04 ^a^ | 2.63±0.21 ^d^ | 0.64±0.05 ^b^ |
| **C3** | 24 | F | 44927 | 300.63±8.94 | 55.63±1.84 ^d^ | 0.87±0.02 ^c^ | 2.56±0.11 ^d^ | 0.64±0.02 ^b^ |
| **C4** | 24 | F | 57465 | 292.84±5.03 | 64.39±2.1 ^d^ | 0.75±0.06 ^c^ | 2.22±0.19 ^c^ | 0.53±0.05 ^b^ |
| **C5** | 18 | M | 36469 | 280.92±7.34 | 61.62±1.76 ^d^ | 0.91±0.02 ^b^ | 2.96±0.14 ^c^ | 0.72±0.03 ^b^ |
| **C6** | 25 | F | 36012 | 301.47±2.56 | 70.85±0.54 ^b^ | 0.91±0.02 ^a^ | 2.97±0.1 ^c^ | 0.7±0.02 ^b^ |
| **C7** | 18 | F | 31802 | 306.7±9.72 | 56.3±0.3 ^d^ | 0.93±0 ^b^ | 3.07±0.04 ^c^ | 0.76±0.01 ^a^ |
| **C8** | 30 | F | 42430 | 304.58±7.41 | 67.97±1.3 ^d^ 8 | 0.93±0.01 ^b^ | 3.14±0.07 ^c^ | 0.74±0.01 ^b^ |
| **C9** | 30 | F | 27857 | 265.94±2.54 | 62.65±2.73 ^b^ | 0.94±0 ^a^ | 3.22±0.02 ^a^ | 0.78±0.01 ^a^ |
| **C10** | 35 | F | 59253 | 253.01±3.7 | 55.18±1.59 ^d^ | 0.9±0.01 ^a^ | 2.77±0.16 ^c^ | 0.69±0.04 ^b^ |
| **C11** | 62 | M | 37700 | 268.99±0.78 | 57.3±1.76 ^d^ | 0.91±0.01 ^a^ | 2.86±0.06 ^c^ | 0.71±0.01 ^b^ |
| **C12** | 30 | M | 11777 | 278.03±7.11 | 63±2.52 ^d^ | 0.92±0 ^b^ | 3.09±0.03 ^b^ | 0.75±0.01 ^a^ |
| **C13** | 50 | M | 23497 | 318.02±16.37 | 57.57±11 .4 ^d^ | 0.73±0.1 ^c^ | 2.1±0.38 ^a^ | 0.52±0.07 ^b^ |
| **C14** | 42 | M | 35906 | 263.91±2.93 | 75±0.58 ^d^ | 0.93±0.01 ^b^ | 3.24±0.07 ^d^ | 0.75±0.02 ^a^ |
| **C15** | 35 | M | 28352 | 312.86±2.96 | 52.72±1.88 ^a^ | 0.75±0.01 ^c^ | 1.89±0.03 ^b^ | 0.48±0.01 ^b^ |
| **C16** | 32 | M | 40189 | 293.44±1.64 | 69.49±1.95 ^b^ | 0.89±0.01 ^b^ | 2.86±0.05 ^c^ | 0.67±0.01 ^b^ |
| **C17** | 31 | M | 12749 | 334.26±12.34 | 37.24±2.71 ^a^ | 0.56±0.06 ^b^ | 1.49±0.21 ^b^ | 0.41±0.05 ^b^ |
| **C18** | 62 | M | 24558 | 331.07±12.22 | 52.22±9.76 ^a^ | 0.65±0.1 ^c^ | 1.67±0.32 ^b^ | 0.42±0.06 ^b^ |
|  |  |  |  |  |  |  |  |  |

**Table S2**. Mean relative abundances of the twelve most abundant bacteria at genus level present in the faecal specimens of 40 kidney-transplant patients and 18 control subjects.

| Genera | Relative abundance (Mean ±SE) | |
| --- | --- | --- |
|  | **Control** | **KT** |
| *Ruminococcaceae.UCG.002* | 2.44±0.25 | 0.77±0.13 |
| *Subdoligranulum* | 1.96±0.36 | 1.25±0.18 |
| *Clostridium.sensu.stricto.1* | 3.44±1.19 | 0.51±0.15 |
| *Dialister* | 2.72±0.46 | 1.38±0.22 |
| *Parabacteroides* | 2.19±0.18 | 1.92±0.23 |
| *Escherichia.Shigella* | 1.01±0.14 | 4.58±0.92 |
| *Roseburia* | 2.69±0.18 | 3.47±0.44 |
| *Alistipes* | 4.75±0.66 | 1.69±0.24 |
| *Succinivibrio* | 2.47±0.58 | 4.79±0.97 |
| *Faecalibacterium* | 7.44±0.64 | 10.7±1.24 |
| *Prevotella.9* | 22.69±2.52 | 14.65±1.95 |
| *Bacteroides* | 22.6±1.84 | 26.74±2.2 |

**Table S3**. Mean relative abundances of the twelve most abundant bacteria at genus level present in faecal specimens of patients receiving a kidney graft over time and of healthy individuals: kidney graft before short (1 year; n= 11); medium-length (2 to 10 years; n=20) and long (>10 years; n=9) periods and in 18 control subjects.

| Genera | Transplantation period subgroups | | | |
| --- | --- | --- | --- | --- |
|  | **Control** | **Short**  **SG** | **Medium MG** | **Long**  **LG** |
| *Rikenellaceae.RC9.gut.group* | 0.98±0.26 | 0.74±0.37 | 0.31±0.17 | 4.04±1.53 |
| *Asteroleplasma* | 0.08±0.05 | 2.56±0.9 | 1.95±1.03 | 2.18±0.53 |
| *Dialister* | 2.72±0.65 | 0.95±0.21 | 0.94±0.21 | 2.9±0.47 |
| *Parabacteroides* | 2.19±0.25 | 1.87±0.27 | 1.69±0.32 | 2.5±0.36 |
| *Sutterella* | 0.26±0.08 | 2.28±0.59 | 0.93±0.31 | 5.91±2.05 |
| *Alistipes* | 4.75±0.94 | 1.35±0.28 | 1.79±0.33 | 1.91±0.43 |
| *Roseburia* | 2.69±0.25 | 5.56±0.81 | 2.70±0.48 | 2.65±0.57 |
| *Escherichia.Shigella* | 1.01±0.2 | 3.11±1.08 | 4.67±1.22 | 6.16±1.70 |
| *Succinivibrio* | 2.47±0.82 | 1.04±0.32 | 3.95±1.10 | 11.24±2.17 |
| *Faecalibacterium* | 7.44±0.91 | 12.15±2.1 | 10.53±1.77 | 9.31±1.24 |
| *Prevotella*.9 | 22.69±3.56 | 16.87±3.34 | 15.91±2.85 | 9.13±1.45 |
| *Bacteroides* | 22.6±2.61 | 32.37±3.3 | 24.29±3.36 | 25.32±2.12 |

**Table S4**. Mean relative abundances of the twelve most abundant bacteria at genus level present in faecal specimens of kidney graft recipients suffering or not from associated diseases: (AD; n=24, no AD; n=16). Associated diseases to kidney graft (AD) in the present study cohort are one or combination of multiple lifestyle diseases: Obesity, diabetes, dyslipidemia, high blood pressure.

| Genera |  | Associated disease (AD) | |
| --- | --- | --- | --- |
|  | **Control** | **no AD** | **AD** |
| *Clostridium.sensu. stricto.1* | 3.44±1.54 | 0.14±0.08 | 0.75±0.25 |
| *Asteroleplasma* | 0.08±0.05 | 2.83±1.08 | 1.73±0.59 |
| *Sutterella* | 0.26±0.07 | 1.28±0.46 | 3.18±1.19 |
| *Dialister* | 2.72±0.59 | 1.29±0.22 | 1.44±0.31 |
| *Parabacteroides* | 2.19±0.23 | 2.71±0.35 | 1.4±0.22 |
| *Alistipes* | 4.75±0.86 | 2.14±0.38 | 1.4±0.26 |
| *Roseburia* | 2.69±0.23 | 3.58±0.61 | 3.4±0.55 |
| *Escherichia.Shigella* | 1.01±0.18 | 6.36±1.48 | 3.39±0.94 |
| *Succinivibrio* | 2.47±0.75 | 4.06±1.20 | 5.28±1.30 |
| *Faecalibacterium* | 7.44±0.83 | 10.83±1.75 | 10.61±1.51 |
| *Prevotella.9* | 22.69±3.25 | 11.75±2.26 | 16.58±2.67 |
| *Bacteroides* | 22.6±2.38 | 34.92±2.99 | 21.29±2.56 |

**Figure S1.** Comparison of the mean relative abundance of bacterial operational taxonomic units (OTUs) at phylum (taxonomic level L2) between study groups: **(A)** control group vs kidney transplant recipients, **(B)** control subjects and patients after short, medium and long post graft-period and **(C)** control subjects, patients suffering and non-suffering from associated diseases.

**Figure S2.** Serial group comparison analysis. Box plot of significantly different OTUs between control and KT individuals (Wilcoxon rank sum test).

**Figure S3.** Serial group comparison analysis. Box plot of significantly different OTU between patients groups over time and healthy individuals: patients receiving a kidney graft before short (1 year; n= 11); medium-length (2 to 10 years; n=20) and long (>10 years; n=9) periods and 18 control subjects (Wilcoxon rank sum test).

**Figure S4.** Serial group comparison analysis. Box plot of significantly different OTU between kidney graft recipients suffering and not suffering from lifestyle diseases: (AD) (n= 24); and no AD (n=16) (Wilcoxon rank sum test).

**Table S5**. Topological properties of networks in fecal communities of healthy and kidney transplant samples.

| Groups | Control | KT | no AD | AD |
| --- | --- | --- | --- | --- |
| Number of nodes | 226 | 227 | 230 | 234 |
| Number of edges | 1417 | 894 | 1704 | 1130 |
| Number of positive interactions /copresence | 498 | 528 | 759 | 629 |
| Number of negative interactions/ mutual exclusion | 775 | 335 | 893 | 468 |
| Clustering coefficient | 0.245 | 0.177 | 0.282 | 0.234 |

**
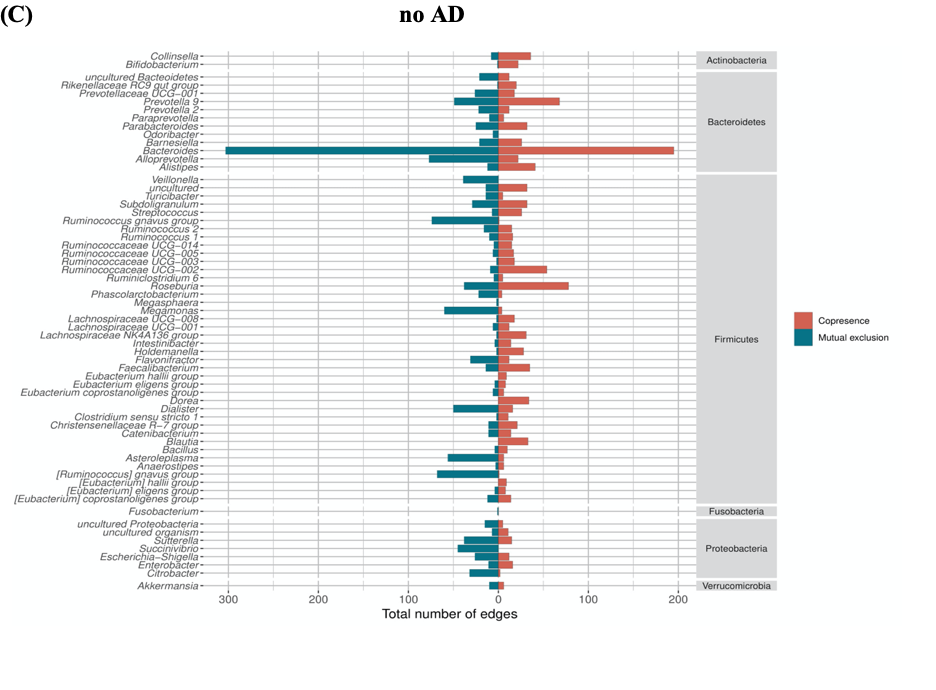
**

**
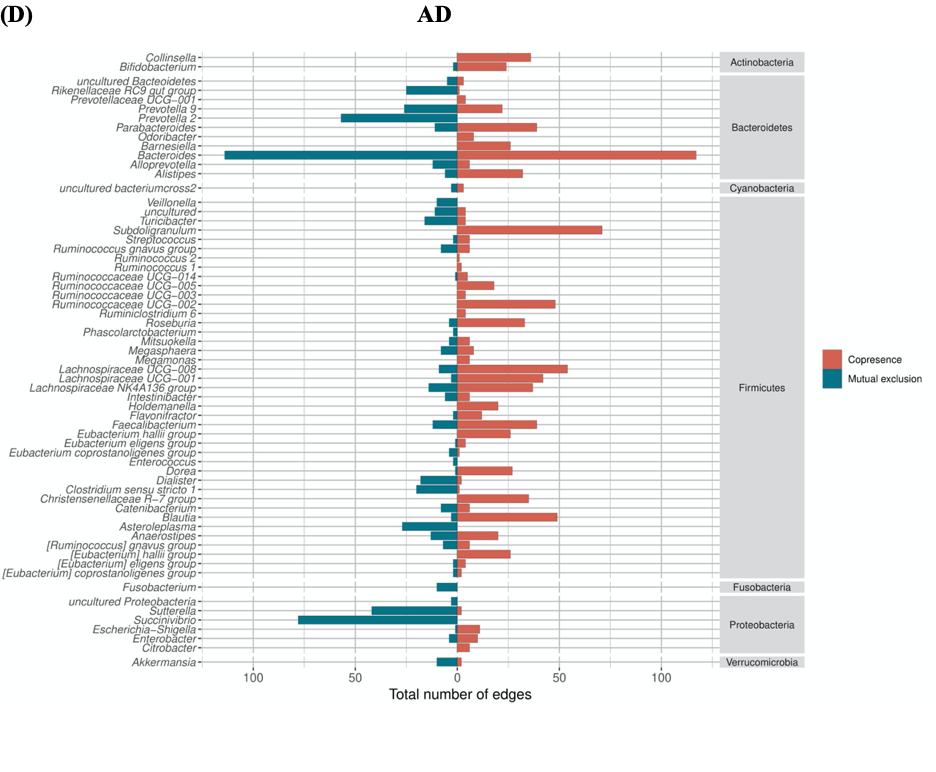
Figure S5.** Total number of positive (Co-presence/Co-occurrence) and negative (Mutual exclusion) relationships between nodes at Genus level within study groups (**A)** Control subjects**,** **(B)** Kidney transplant recipients**, (C)** Patients with no associated diseases**,** and (**D)** AD group.
