# Supplementary material for: Impact of the Post-Transplant Period and Lifestyle Diseases on Human Gut Microbiota in Kidney Graft Recipients
Source: Microorganisms. 2020 Nov 4;8(11):1724. doi: 10.3390/microorganisms8111724 (PMC7694191; doi:10.3390/microorganisms8111724)
Supplement: Supplementary file 1 [file microorganisms-08-01724-s001.zip › Table S1.docx]

**Table S1**. Richness and diversity estimation of the 16S *rRNA* gene libraries of kidney transplanted patients through the amplicon sequence analysis. (M: male, F: female), Asterisks indicates groups similarity calculated by Student t-test, *P*<.05. Significant differences are indicated by different letters.

|  | **Age** | **Gender** | **Number of Reads** | **OTU number** | **Richness** | **Simpson** | **Shannon** | **Pielous**  **evenness** |
| --- | --- | --- | --- | --- | --- | --- | --- | --- |
| **P1** | 21 | M | 6935 | 295.09±5.27 | 44.33±0.33^a^ | 0.9±0.002 ^a^ | 2.77±0.01 ^a^ | 0.73±0.004 ^a^ |
| **P2** | 53 | M | 13615 | 326.11±3.86 | 37.67±3.71^a^ | 0.77±0.02 ^b^ | 2.01±0.06 ^b^ | 0.56±0.03 ^b^ |
| **P3** | 35 | F | 3883 | 324.33±1.63 | 63±3.21^b^ | 0.92±0.01 ^b^ | 3.05±0.05 ^c^ | 0.74±0.01 ^a^ |
| **P4** | 47 | M | 3829 | 338.66±0.19 | 45.67±4.06^c^ | 0.67±0.01 ^c^ | 1.93±0.04 ^b^ | 0.51±0.01 ^b^ |
| **P5** | 67 | M | 1768 | 282.1±3.46 | 51.67±6.01^a^ | 0.9±0.01 ^a^ | 2.98±0.1 ^c^ | 0.76±0.006 ^a^ |
| **P6** | 37 | M | 13210 | 310.83±4.26 | 57.28±7.14^d^ | 0.86±0.009 ^a^ | 2.54±0.06 ^d^ | 0.63±0.04 ^b^ |
| **P7** | 37 | M | 17736 | 314.14±1.66 | 57.28±2.29^d^ | 0.89±0.003 ^a^ | 2.72±0.03 ^d^ | 0.67±0.01 ^b^ |
| **P8** | 66 | M | 12803 | 304.67±7.05 | 64.99±3.05^d^ | 0.92±0.01 ^b^ | 2.98±0.07 ^c^ | 0.71±0.02 ^b^ |
| **P9** | 48 | F | 13410 | 323.41±15.62 | 29.28±4.02^d^ | 0.3±0.1 ^d^ | 0.78±0.23 ^e^ | 0.23±0.06 ^c^ |
| **P10** | 18 | F | 17780 | 331.34±0.56 | 34.97±4.14^a^ | 0.79±0.01 ^b^ | 1.98±0.03 ^b^ | 0.56±0.02 ^b^ |
| **P11** | 29 | M | 15986 | 317.02±1.88 | 64.64±0.87^d^ | 0.82±0.01 ^b^ | 2.47±0.03 ^d^ | 0.59±0.01 ^b^ |
| **P12** | 58 | M | 26812 | 255.44±8.84 | 63.66±5.36^d^ | 0.93±0.01 ^b^ | 3.01±0.09 ^c^ | 0.73±0.01 ^a^ |
| **P13** | 33 | F | 17208 | 340.21±2.52 | 53.56±5.4^a^ | 0.74±0.02 ^c^ | 2.05±0.04 ^b^ | 0.52±0.01 ^b^ |
| **P14** | 56 | M | 15223 | 328.69±2.27 | 54.3±2.3^a^ | 0.77±0.01 ^b^ | 2.01±0.09 ^b^ | 0.5±0.03 ^b^ |
| **P15** | 39 | M | 13777 | 301.7±3.05 | 55.97±11.49^d^ | 0.93±0.01 ^b^ | 3.02±0.04 ^c^ | 0.77±0.06 ^a^ |
| **P16** | 57 | M | 16283 | 268.42±16.63 | 65.67±3.84 ^d^ | 0.85±0.08 ^a^ | 2.9±0.4 ^c^ | 0.69±0.09 ^b^ |
| **P17** | 53 | M | 1755 | 299.22±3.59 | 29.67±2.85 ^d^ | 0.75±0.07 ^c^ | 1.99±0.27 ^b^ | 0.6±0.1 ^b^ |
| **P18** | 51 | M | 10328 | 283.12±3.45 | 65±0.58 ^d^ | 0.92±0.01 ^b^ | 3.09±0.05 ^c^ | 0.74±0.01 ^a^ |
| **P19** | 46 | M | 12513 | 322.73±7.05 | 53.66±3.84 ^a^ | 0.83±0.07 ^b^ | 2.54±0.29 ^d^ | 0.64±0.06 ^b^ |
| **P20** | 36 | F | 7399 | 315.23±4.28 | 27.67±1.86 ^d^ | 0.69±0.04 ^c^ | 1.73±0.13 ^b^ | 0.52±0.03 ^b^ |
| **P21** | 60 | F | 12115 | 331.8±3.9 | 50.66±2.34 ^a^ | 0.56±0.12 ^b^ | 1.56±0.33 ^b^ | 0.4±0.08 ^b^ |
| **P22** | 17 | M | 9962 | 308.17±4.76 | 46.66±0.88 ^c^ | 0.85±0.03 ^a^ | 2.54±0.14 ^d^ | 0.66±0.03 ^b^ |
| **P23** | 26 | M | 14075 | 333.82±10.9 | 35.65±0.88 ^a^ | 0.73±0.02 ^c^ | 1.78±0.11 ^b^ | 0.5±0.03 ^b^ |
| **P24** | 42 | F | 16675 | 323.93±12.15 | 49.58±9.82 ^a^ | 0.81±0.04 ^b^ | 2.3±0.24 ^b^ | 0.59±0.03 ^b^ |
| **P25** | 43 | F | 22664 | 333.07±2.2 | 60.12±2.16 ^d^ | 0.81±0.01 ^b^ | 2.35±0.1 ^b^ | 0.57±0.02 ^b^ |
| **P26** | 42 | F | 14568 | 308.01±4.79 | 59.33±1.76 ^d^ | 0.81±0.02 ^b^ | 2.45±0.06 ^d^ | 0.6±0.02 ^b^ |
| **P27** | 50 | F | 14450 | 325.05±20.47 | 51.33±8.84 ^a^ | 0.68±0.1 ^c^ | 2.06±0.36 ^b^ | 0.52±0.07 ^b^ |
| **P28** | 41 | M | 22288 | 320.42±14.03 | 64.22±4.21 ^d^ | 0.8±0.11 ^b^ | 2.55±0.39 ^d^ | 0.61±0.09 ^b^ |
| **P29** | 27 | M | 21007 | 312.23±6.97 | 66.63±2.34 ^d^ | 0.7±0.02 ^c^ | 2.17±0.09 ^b^ | 0.52±0.02 ^b^ |
| **P30** | 61 | M | 15217 | 231.8±2.54 | 56.33±1.86 ^d^ | 0.83±0.03 ^b^ | 2.5±0.1 ^d^ | 0.62±0.02 ^b^ |
| **P31** | 43 | F | 29035 | 345.23±5.3 | 34.98±1.08 ^a^ | 0.8±0.02 ^b^ | 1.94±0.09 ^b^ | 0.54±0.03 ^b^ |
| **P32** | 37 | M | 19795 | 339.4±12.54 | 61.33±3.59 ^d^ | 0.58±0.2 ^b^ | 1.85±0.65 ^b^ | 0.45±0.16 ^b^ |
| **P33** | 30 | M | 15420 | 327.39±4.58 | 29.66±1.2 ^d^ | 0.71±0.01 ^c^ | 1.74±0.03 ^b^ | 0.51±0 ^b^ |
| **P34** | 50 | M | 21069 | 310.85±2.38 | 60.97±2.06 ^d^ | 0.78±0.01 ^b^ | 2.43±0.06 ^b^ | 0.59±0.01 ^b^ |
| **P35** | 41 | M | 10042 | 338.47±5.86 | 28.27±1.97 ^d^ | 0.73±0.08 ^c^ | 1.76±0.46 ^d^ | 0.53±0.15 ^b^ |
| **P36** | 30 | M | 12602 | 318.01±4.05 | 49.33±3.29 ^a^ | 0.85±0.01 ^a^ | 2.55±0.09 ^d^ | 0.66±0.02 ^b^ |
| **P37** | 39 | M | 47005 | 308.19±2.32 | 49.66±1.22 ^a^ | 0.86±0.02 ^a^ | 2.53±0.15 ^c^ | 0.64±0.04 ^b^ |
| **P38** | 47 | M | 42892 | 325.16±7.68 | 62.94±7.99 ^b^ | 0.92±0.01 ^b^ | 2.99±0.21 ^c^ | 0.72±0.03 ^b^ |
| **P39** | 42 | F | 72013 | 334.21±4.72 | 64.6±2.58 ^d^ | 0.9±0.01 ^a^ | 2.87±0.12 ^b^ | 0.69±0.02 ^b^ |
| **P40** | 44 | M | 21589 | 371.64±0.38 | 24.68±4.55 ^d^ | 0.16±0.03 ^d^ | 0.38±0.06 ^b^ | 0.12±0.01 ^b^ |
| **C1** | 24 | F | 35517 | 278.76±5.01 | 66.92±3.6 ^d^ | 0.72±0.06 ^c^ | 2.18±0.34 ^d^ | 0.52±0.08 ^b^ |
| **C2** | 24 | F | 22288 | 299.5±1.6 | 60.58±6.32 ^d^ | 0.84±0.04 ^a^ | 2.63±0.21 ^d^ | 0.64±0.05 ^b^ |
| **C3** | 24 | F | 44927 | 300.63±8.94 | 55.63±1.84 ^d^ | 0.87±0.02 ^c^ | 2.56±0.11 ^d^ | 0.64±0.02 ^b^ |
| **C4** | 24 | F | 57465 | 292.84±5.03 | 64.39±2.1 ^d^ | 0.75±0.06 ^c^ | 2.22±0.19 ^c^ | 0.53±0.05 ^b^ |
| **C5** | 18 | M | 36469 | 280.92±7.34 | 61.62±1.76 ^d^ | 0.91±0.02 ^b^ | 2.96±0.14 ^c^ | 0.72±0.03 ^b^ |
| **C6** | 25 | F | 36012 | 301.47±2.56 | 70.85±0.54 ^b^ | 0.91±0.02 ^a^ | 2.97±0.1 ^c^ | 0.7±0.02 ^b^ |
| **C7** | 18 | F | 31802 | 306.7±9.72 | 56.3±0.3 ^d^ | 0.93±0 ^b^ | 3.07±0.04 ^c^ | 0.76±0.01 ^a^ |
| **C8** | 30 | F | 42430 | 304.58±7.41 | 67.97±1.3 ^d^ 8 | 0.93±0.01 ^b^ | 3.14±0.07 ^c^ | 0.74±0.01 ^b^ |
| **C9** | 30 | F | 27857 | 265.94±2.54 | 62.65±2.73 ^b^ | 0.94±0 ^a^ | 3.22±0.02 ^a^ | 0.78±0.01 ^a^ |
| **C10** | 35 | F | 59253 | 253.01±3.7 | 55.18±1.59 ^d^ | 0.9±0.01 ^a^ | 2.77±0.16 ^c^ | 0.69±0.04 ^b^ |
| **C11** | 62 | M | 37700 | 268.99±0.78 | 57.3±1.76 ^d^ | 0.91±0.01 ^a^ | 2.86±0.06 ^c^ | 0.71±0.01 ^b^ |
| **C12** | 30 | M | 11777 | 278.03±7.11 | 63±2.52 ^d^ | 0.92±0 ^b^ | 3.09±0.03 ^b^ | 0.75±0.01 ^a^ |
| **C13** | 50 | M | 23497 | 318.02±16.37 | 57.57±11 .4 ^d^ | 0.73±0.1 ^c^ | 2.1±0.38 ^a^ | 0.52±0.07 ^b^ |
| **C14** | 42 | M | 35906 | 263.91±2.93 | 75±0.58 ^d^ | 0.93±0.01 ^b^ | 3.24±0.07 ^d^ | 0.75±0.02 ^a^ |
| **C15** | 35 | M | 28352 | 312.86±2.96 | 52.72±1.88 ^a^ | 0.75±0.01 ^c^ | 1.89±0.03 ^b^ | 0.48±0.01 ^b^ |
| **C16** | 32 | M | 40189 | 293.44±1.64 | 69.49±1.95 ^b^ | 0.89±0.01 ^b^ | 2.86±0.05 ^c^ | 0.67±0.01 ^b^ |
| **C17** | 31 | M | 12749 | 334.26±12.34 | 37.24±2.71 ^a^ | 0.56±0.06 ^b^ | 1.49±0.21 ^b^ | 0.41±0.05 ^b^ |
| **C18** | 62 | M | 24558 | 331.07±12.22 | 52.22±9.76 ^a^ | 0.65±0.1 ^c^ | 1.67±0.32 ^b^ | 0.42±0.06 ^b^ |
|  |  |  |  |  |  |  |  |  |
