# Supplementary material for: Impact of the Post-Transplant Period and Lifestyle Diseases on Human Gut Microbiota in Kidney Graft Recipients
Source: Microorganisms. 2020 Nov 4;8(11):1724. doi: 10.3390/microorganisms8111724 (PMC7694191; doi:10.3390/microorganisms8111724)
Supplement: Supplementary file 1 [file microorganisms-08-01724-s001.zip › Table S2.docx]

**Table S2**. Mean relative abundances of the twelve most abundant bacteria at genus level present in the faecal specimens of 40 kidney-transplant patients and 18 control subjects.

| Genera | Relative abundance (Mean ±SE) | |
| --- | --- | --- |
|  | **Control** | **KT** |
| *Ruminococcaceae.UCG.002* | 2.44±0.25 | 0.77±0.13 |
| *Subdoligranulum* | 1.96±0.36 | 1.25±0.18 |
| *Clostridium.sensu.stricto.1* | 3.44±1.19 | 0.51±0.15 |
| *Dialister* | 2.72±0.46 | 1.38±0.22 |
| *Parabacteroides* | 2.19±0.18 | 1.92±0.23 |
| *Escherichia.Shigella* | 1.01±0.14 | 4.58±0.92 |
| *Roseburia* | 2.69±0.18 | 3.47±0.44 |
| *Alistipes* | 4.75±0.66 | 1.69±0.24 |
| *Succinivibrio* | 2.47±0.58 | 4.79±0.97 |
| *Faecalibacterium* | 7.44±0.64 | 10.7±1.24 |
| *Prevotella.9* | 22.69±2.52 | 14.65±1.95 |
| *Bacteroides* | 22.6±1.84 | 26.74±2.2 |
