# Supplementary material for: Impact of the Post-Transplant Period and Lifestyle Diseases on Human Gut Microbiota in Kidney Graft Recipients
Source: Microorganisms. 2020 Nov 4;8(11):1724. doi: 10.3390/microorganisms8111724 (PMC7694191; doi:10.3390/microorganisms8111724)
Supplement: Supplementary file 1 [file microorganisms-08-01724-s001.zip › Table S3.docx]

**Table S3**. Mean relative abundances of the twelve most abundant bacteria at genus level present in faecal specimens of patients receiving a kidney graft over time and of healthy individuals: kidney graft before short (1 year; n= 11); medium-length (2 to 10 years; n=20) and long (>10 years; n=9) periods and in 18 control subjects.

| Genera | Transplantation period subgroups | | | |
| --- | --- | --- | --- | --- |
|  | **Control** | **Short**  **SG** | **Medium MG** | **Long**  **LG** |
| *Rikenellaceae.RC9.gut.group* | 0.98±0.26 | 0.74±0.37 | 0.31±0.17 | 4.04±1.53 |
| *Asteroleplasma* | 0.08±0.05 | 2.56±0.9 | 1.95±1.03 | 2.18±0.53 |
| *Dialister* | 2.72±0.65 | 0.95±0.21 | 0.94±0.21 | 2.9±0.47 |
| *Parabacteroides* | 2.19±0.25 | 1.87±0.27 | 1.69±0.32 | 2.5±0.36 |
| *Sutterella* | 0.26±0.08 | 2.28±0.59 | 0.93±0.31 | 5.91±2.05 |
| *Alistipes* | 4.75±0.94 | 1.35±0.28 | 1.79±0.33 | 1.91±0.43 |
| *Roseburia* | 2.69±0.25 | 5.56±0.81 | 2.70±0.48 | 2.65±0.57 |
| *Escherichia.Shigella* | 1.01±0.2 | 3.11±1.08 | 4.67±1.22 | 6.16±1.70 |
| *Succinivibrio* | 2.47±0.82 | 1.04±0.32 | 3.95±1.10 | 11.24±2.17 |
| *Faecalibacterium* | 7.44±0.91 | 12.15±2.1 | 10.53±1.77 | 9.31±1.24 |
| *Prevotella*.9 | 22.69±3.56 | 16.87±3.34 | 15.91±2.85 | 9.13±1.45 |
| *Bacteroides* | 22.6±2.61 | 32.37±3.3 | 24.29±3.36 | 25.32±2.12 |
