# Supplementary material for: Impact of the Post-Transplant Period and Lifestyle Diseases on Human Gut Microbiota in Kidney Graft Recipients
Source: Microorganisms. 2020 Nov 4;8(11):1724. doi: 10.3390/microorganisms8111724 (PMC7694191; doi:10.3390/microorganisms8111724)
Supplement: Supplementary file 1 [file microorganisms-08-01724-s001.zip › Table S4.docx]

**Table S4**. Mean relative abundances of the twelve most abundant bacteria at genus level present in faecal specimens of kidney graft recipients suffering or not from associated diseases: (AD; n=24, no AD; n=16). Associated diseases to kidney graft (AD) in the present study cohort are one or combination of multiple lifestyle diseases: Obesity, diabetes, dyslipidemia, high blood pressure.

| Genera |  | Associated disease (AD) | |
| --- | --- | --- | --- |
|  | **Control** | **no AD** | **AD** |
| *Clostridium.sensu. stricto.1* | 3.44±1.54 | 0.14±0.08 | 0.75±0.25 |
| *Asteroleplasma* | 0.08±0.05 | 2.83±1.08 | 1.73±0.59 |
| *Sutterella* | 0.26±0.07 | 1.28±0.46 | 3.18±1.19 |
| *Dialister* | 2.72±0.59 | 1.29±0.22 | 1.44±0.31 |
| *Parabacteroides* | 2.19±0.23 | 2.71±0.35 | 1.4±0.22 |
| *Alistipes* | 4.75±0.86 | 2.14±0.38 | 1.4±0.26 |
| *Roseburia* | 2.69±0.23 | 3.58±0.61 | 3.4±0.55 |
| *Escherichia.Shigella* | 1.01±0.18 | 6.36±1.48 | 3.39±0.94 |
| *Succinivibrio* | 2.47±0.75 | 4.06±1.20 | 5.28±1.30 |
| *Faecalibacterium* | 7.44±0.83 | 10.83±1.75 | 10.61±1.51 |
| *Prevotella.9* | 22.69±3.25 | 11.75±2.26 | 16.58±2.67 |
| *Bacteroides* | 22.6±2.38 | 34.92±2.99 | 21.29±2.56 |
