# Supplementary material for: Impact of the Post-Transplant Period and Lifestyle Diseases on Human Gut Microbiota in Kidney Graft Recipients
Source: Microorganisms. 2020 Nov 4;8(11):1724. doi: 10.3390/microorganisms8111724 (PMC7694191; doi:10.3390/microorganisms8111724)
Supplement: Supplementary file 1 [file microorganisms-08-01724-s001.zip › Table S5.docx]

**Table S5**. Topological properties of networks in fecal communities of healthy and kidney transplant samples.

| Groups | Control | KT | no AD | AD |
| --- | --- | --- | --- | --- |
| Number of nodes | 226 | 227 | 230 | 234 |
| Number of edges | 1417 | 894 | 1704 | 1130 |
| Number of positive interactions /copresence | 498 | 528 | 759 | 629 |
| Number of negative interactions/ mutual exclusion | 775 | 335 | 893 | 468 |
| Clustering coefficient | 0.245 | 0.177 | 0.282 | 0.234 |
